# Evaluation of water-economy-ecology system development level and coupling coordination degree: a case study of China’s central plains urban agglomeration

**DOI:** 10.1038/s41598-026-44489-2

**Published:** 2026-03-20

**Authors:** Huan Yang, Jiahao Shi, Cheng Lü, Fuqiang Wang, Peiheng Liu, Subing Lü, Jiageng Hu

**Affiliations:** 1 North China University of Water Resources and Electric Power, Zhengzhou, 450046 China; 2 Henan Key Laboratory of Water Resources Conservation and Intensive Utilization in the Yellow River Basin, Zhengzhou, 450046 China; 3 Song-Liao Water Resources Commission Basin Planning & Policy Research Center, Changchun, 130021 China; 4 Tonghua Water Conservancy Bureau, Tonghua, 134000 China

**Keywords:** Water-economy-ecology (WEE) system, Improved coupling coordination degree model (ICCDM), EFAST-cloud model (ECM), Spatial connection analysis, Central plains urban agglomeration (CPUA), Ecology, Ecology, Environmental sciences, Environmental social sciences

## Abstract

The water-economy-ecology (WEE) system exhibits a complex coupling relationship, presenting significant challenges to sustainable urban agglomeration development. This study proposes a novel evaluation framework to explore the coupling coordination degree (CCD) of the regional WEE system for sustainable urban agglomeration development. By integrating the EFAST-Cloud model (ECM) to quantify the comprehensive development level of each subsystem, and applying an improved coupling coordination degree model (ICCDM), the spatio-temporal evolution of CCD in China’s Central plains urban agglomeration (CPUA) was analyzed. Results indicate: (1) The comprehensive evaluation index (CDI) of the WEE system shows an upward trend, with Xinyang, Zhengzhou, Huaibei, and Jiyuan in the leading position in their respective subsystems and the WEE system. (2) From 2011 to 2020, CCD exhibited a fluctuating but increasing trend, with the dominant coupling relationship shifting from water-economy to water-ecology. The core development area (CDA) consistently outperformed the non-core area (NCDA), with Jiyuan, Huaibei, and Zhengzhou achieving the highest CCD values. (3) Spatial analysis indicated a gradual strengthening of global spatial autocorrelation, while local autocorrelation was dominated by L-L and H-L clusters with limited spatial extent. The gravitational effect of CCD became increasingly pronounced by 2020, with Zhengzhou consistently emerging as the dominant center of gravity (COG) for CCD distribution. This study could not only provide a robust methodological framework for WEE system research but also offer new theoretical and practical insights into sustainable development in urban agglomerations.

## Introduction

The WEE system constitutes an organic complex formed through the interconnection, interaction, and coupling of water, economic, and ecological subsystems. Within this integrated system, continuous material cycles, energy flows, and information exchanges occur, establishing complex dynamic feedback mechanisms among system components^1,2^. Achieving WEE system coordination, mitigating water resource pressures, and maintaining ecological balance while supporting sustainable socioeconomic development are critical issues at both regional and global scales. Therefore, quantitative research on the interactions and coordinated development degrees among internal elements of the WEE system is essential for the scientific management of water resources.

CCD serves as a crucial metric for evaluating both the interdependence among system components and their coordinated development status, representing a vital parameter that reflects a system’s integrity and harmonious functioning. Early studies about CCD were mainly appeared in engineering, and then gradually introduced social-economic development and water environment^3^, water-energy-food and poverty^4^, urbanization and flood disasters^1,2^, sustainable development and ecosystem services^5^. Given the intricate and interconnected relationships among water resources, the economy and society, and the ecological environment, conducting systematic research by treating them as an integrated whole is conducive to uncovering the coordinated development dynamics within this complex system and providing a theoretical foundation for scientific regulation and control. In recent years, some scholars have begun to apply CCD to the study of WEE system, but the initially focused on the relationship between economic society and ecological environment. With the research developed, some researchers tried to join the third-party system to analyze the composite relationship of WEE. Yan et al.^6^ and Lu et al.^7^ validate the necessity of coordinated development by exploring the matching degree of water-economy-ecosystems and the intrinsic mechanism. Zhou et al.^8^ conducted a scientific evaluation of the water-economy-ecosystem (WSE) in Anhui Province, China, by building a model based on connection numbers for the purpose of coordinating development evaluation^9–11^ constructed a diagnostic framework for the spatial balance of water resources considering WEE nexus, and carried out an in-depth study on the spatial balance of water resources. Hu et al.^12^ analyzed the dynamic evolution of coupling coordination in the WEE system of the CPUA, optimized coordination-level classification, and predicted future development trends under multiple scenarios.

In this context, the formation and coordinated development of urban agglomerations have emerged as a new research focus. An urban agglomeration refers to a group of cities formed within a specific geographical area with a mega city as its core, at least three or more metropolitan areas (districts) or large cities as its basic constituent units, and relying on a well-developed network of transportation and communications with a compact spatial organization, close economic cities, and eventual co-location and a high degree of integration^13^. Urban agglomerations are the inevitable product of the development of cities from competition to cooperation, are highly integrated city groups, and they are an important bearing place for the transfer of the center of gravity of the world economy. In China’s eight national-level urban agglomerations, the CPUA serves as a new growth pole for the country’s economic development, holding significant economic, transportation, and strategic importance. The water resources of CPUA are short and mainly concentrated in flood season, and the water resources of each city vary greatly^14^, its development is crucial not only to the economic growth of Central China but also plays a significant role in the national socioeconomic advancement.

Despite the growing body of research on WEE coupling coordination, several critical gaps remain: (1) Limited spatial scale: Most studies focus on single cities or river basins, with few addressing urban agglomerations. (2) Lack of subsystem differentiation: Research tends to focus on the overall CCD of the WEE system, without adequately evaluating the individual development levels of each subsystem or the CCD of dual-system interactions. (3) Insufficient spatial analysis: There is a lack of studies on the spatial relationship of WEE CCD, including spatial autocorrelation, spatial clustering, and dynamic spatial shifts.

Based on the above, this study takes the CPUA as the target area to carry out the coupling coordination evaluation of the WEE system. Firstly, an index system covering 3 criterion layers and 25 indicators was constructed, and the subjective and objective combination method of AHP-EFAST was used to calculate the weights of the indicators. Secondly, the ECM was used to evaluate and analyze the development level of the three subsystems and the WEE composite system. Thirdly, the ICCDM was used to calculate the CCD of dual and composite systems and analyzed on three scales: urban agglomeration, urban partition, and municipal. Finally, the spatial relationship of the CCD of the WEE system in the CPUA is analyzed and discussed in terms of spatial autocorrelation, spatial gravity, and COG migration. The results of the study can provide a scientific basis and theoretical foundation for regional high-quality development, which has enormous practical implications.

## Materials and methods

### Study area and data

**Fig. 1:**
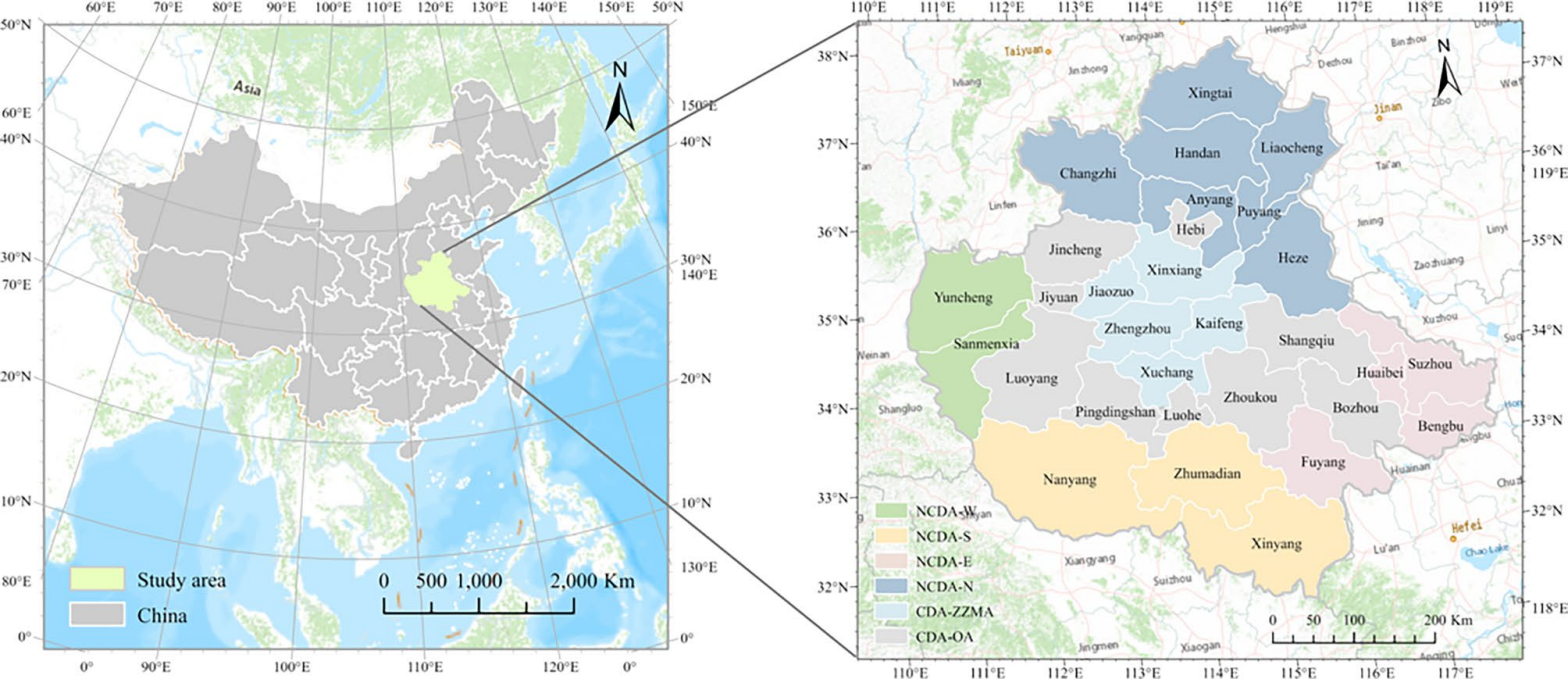
Location of study area- China’s Central Plains Urban Agglomeration (CPUA) (created using ArcGIS 10.8, https://www.esri.com, based on the standard map GS(2019)1822).

The CPUA occupies 287,000 km^2^ in central-eastern China’s Yellow River basin, spanning 30 cities within Henan, Hebei, Shanxi, Shandong, and Anhui Provinces (Fig. 1). Over half (56%) of its area is mountainous, while the remainder consists of hills and lakeside zones. As the cradle of Chinese civilization, the CPUA features a topographical gradient descending from west to east, characterized by a mix of plains and mountainous terrain under a continental monsoon climate. The region is geographically bounded by the Taihang Mountains to the northwest, Funiushan Mountains to the southwest, and Tongbai-Dabie Mountains to the southeast, while its central area comprises the agriculturally vital Huanghuai Plain, a cornerstone of China’s agricultural production. In 2020, the total population of the CPUA reached 163 million, with a per capita GDP of 58,659 yuan and a gross regional product of 81,266 billion yuan, ranking fourth in total GDP among China’s urban agglomerations.

According to China’s CPUA Development Plan, the region is administratively divided into two primary components: the Core Development Area (CDA) and the Non-Core Development Area (NCDA). The CDA encompasses 13 cities, with Zhengzhou, Kaifeng, Xinxiang, Jiaozuo, and Xuchang collectively designated as the Zhengzhou Metropolitan Area (CDA-ZZMA), while the remaining CDA cities constitute Other Urban Areas (CDA-OA). The NCDA comprises four functionally distinct areas: the northern cross-regional synergistic development demonstration area (NCDA-N), the eastern industrial transfer demonstration area (NCDA-E), the southern high-efficiency ecological economy demonstration area (NCDA-S), and the western transformation and innovative development demonstration area (NCDA-W).

The primary dataset for this study is sourced from the official "Water Resources Bulletins" published by the provincial governments of Henan, Hebei, Shanxi, Shandong, and Anhui from 2011 to 2020. These bulletins are publicly accessible through the official websites of the water resources departments of each province. Supplementary data is extracted from: (1) provincial statistical yearbooks from 2010 to 2019; (2) environmental status bulletins from 2011 to 2020; and (3) other government-approved databases. Although minor discrepancies exist in the data due to differences in statistical definitions, and a small number of missing data were addressed using interpolation methods these limitations are unlikely to substantially affect the overall validity of our findings, given the large sample size and consistent trends observed across multiple datasets.

### Index system and thresholds

#### Construction of evaluation index system

The establishment of a scientifically objective evaluation index system constitutes the fundamental prerequisite for assessing regional Water-Economy-Ecology (WEE) coupling coordination in a rigorous and equitable manner. An effective index system should satisfy four essential criteria: (1) conceptual clarity and interpretability, (2) data accessibility, (3) standardized units for cross-spatiotemporal comparability, and (4) substantive relevance to accurately capture systemic essence. Guided by these criteria and adhering to the principles of representativeness, comprehensiveness, practicality, operational feasibility, comparability, and territorial specificity, the index system for evaluating the CCD of CPUA was screened from a large number of indicators and finally determined as Table 1.

**Table 1:** Evaluation index system for the coordination degree of WEE.

Criterion layer	Index layer	Unit	Character	Significance
*A*: Water	*A*_*1*_: Per-capita water resource	m^3^	+	Water resources status
	*A*_*2*_: Precipitation	mm	+	Regional natural water supply
	*A*_*3*_: Water production coefficient	/	+	The precipitation’s capacity to turn into water
	*A*_*4*_: Water production modulus	10000m^3^/km^2^	+	Distribution of total water resources by region
	*A*_*5*_: Per-capita domestic water consumption	m^3^	+	Regional water resources utilization and living standards
	*A*_*6*_: Water consumption for 10,000 yuan of GDP	m^3^	-	Regional economic development level
	*A*_*7*_: Water consumption for 10,000 yuan of industrial added valueindustry	m^3^	-	Industrial water use efficiency
	*A*_*8*_: Average water consumption per mu for farmland irrigation	m^3^	-	Irrigation efficiency and levels
	*A*_*9*_: Utilization rate of water resources development	%	< 1 is + ; > 1 is -	Water resources development and utilization
	*A*_*10*_: Proportion of groundwater supply	%	-	Exploitation and use of groundwater
*B*: Economy	*B*_*1*_: Per-capita GDP	10000yuan	+	Regional economic development level and industrial development
	*B*_*2*_: Population density	person/km^2^	+	Regional population distribution
	*B*_*3*_: Per-capita total industrial output value	10000 yuan	+	The scale and level of industrial production
	*B*_*4*_: Urbanization rate	%	+	Urban and rural distribution of permanent population
	*B*_*5*_: Percentage of water employees	‰	+	Level of development of water resources science and technology
	*B*_*6*_: Percentage of investment in water supply and drainage	%	+	Degree of investment in water resources projects
	*B*_*7*_: Natural rate of population growth	‰	+	The extent and tendency of natural population growth
*C*: Ecology	*C*_*1*_: Greening coverage in built-up areas	%	+	Status of regional ecological and environmental protection
	*C*_*2*_: Proportion of ecological water use	%	+	Regional sewage situation
	*C*_*3*_: Industrial wastewater discharge with an output value of 10,000,000 yuan	m^3^	-	Improvement of water quality and ecological capacity
	*C*_*4*_: Sewage treatment rate	%	-	Status of water resources protection and ecosystems
	*C*_*5*_: Non-hazardous treatment rate of domestic waste	%	+	Municipal waste treatment capacity and environmental protection level
	*C*_*6*_: Drainage pipe density	km	+	Degree of sparsity of drainage pipeline distribution in the region
	*C*_*7*_:Normalized difference vegetation index	/	+	Status of changes in surface vegetation cover
	*C*_*8*_: Proportion of recycled water supply	%	-	Water resources utilization and water ecology virtuous cycle level

#### Classification criteria for evaluation index thresholds

Based on regional field investigations and integrated analysis of relevant studies^12,15–18^, this study establishes a five-tier classification system for evaluation index thresholds, which are excellent, healthy, sub-healthy, unhealthy and sick. The threshold values were determined by integrating empirical regional data with benchmarks established in previous studies, ensuring both local applicability and methodological consistency with existing research. These graded thresholds, corresponding to distinct developmental statuses, serve as benchmarks for evaluating indicator performance. The specific typology and threshold ranges for each indicator level are detailed in Table 2.

**Table 2:** Classification criteria for evaluation index thresholds.

Index number	Excellent	Healthy	Sub-healthy	Unhealthy	Sick
*A*_*1*_	> 500	(400, 500]	(200, 400]	(100, 200]	≤ 100
*A*_*2*_	(1000, 1600]	(750, 1000]	(600, 750]	(500, 600]	≤ 500 or > 1600
*A*_*3*_	> 0.40	(0.30, 0.40]	(0.20, 0.30]	(0.15, 0.20]	≤ 0.15
*A*_*4*_	> 20	(15, 20]	(10, 15]	(5, 10]	≤ 5
*A*_*5*_	> 70	(50, 70]	(40, 50]	(30, 40]	≤ 30
*A*_*6*_	≤ 30	(30, 50]	(50, 100]	(100, 200]	> 200
*A*_*7*_	≤ 15	(15, 25]	(25, 45]	(45, 60]	> 60
*A*_*8*_	≤ 100	(100, 150]	(150, 200]	(200, 300]	> 300
*A*_*9*_	≤ 30	(30, 50]	(50, 70]	(70, 90]	> 90
*A*_*10*_	≤ 25	(25, 40]	(40, 55]	(55, 70]	> 70
*B*_*1*_	> 8	(6, 8]	(3.5, 6]	(2, 3.5]	≤ 2
*B*_*2*_	(1000, 1700]	(800, 1000]	(600, 800]	(400, 600]	≤ 400 or > 1700
*B*_*3*_	> 4	(3, 4]	(1.5, 3]	(1, 1.5]	≤ 1
*B*_*4*_	> 60	(50, 60]	(40, 50]	(30, 40]	≤ 30
*B*_*5*_	> 4	(3, 4]	(2, 3]	(1, 2]	≤ 1
*B*_*6*_	> 40	(20, 40]	(8, 20]	(2, 8]	≤ 2
*B*_*7*_	(12, 25]	(8, 12]	(5, 8]	(1, 5]	≤ 1 or > 25
*C*_*1*_	> 45	(40, 45]	(35, 40]	(30, 35]	≤ 30
*C*_*2*_	> 15	(8, 15]	(3, 8]	(1, 3]	≤ 1
*C*_*3*_	≤ 2	(2, 4]	(4, 8]	(8, 12]	> 12
*C*_*4*_	100	(98, 100]	(95, 98]	(80, 95]	≤ 80
*C*_*5*_	100	(95, 100]	(80, 95]	(70, 80]	≤ 70
*C*_*6*_	> 15	(10, 15]	(7.5, 10]	(5, 7.5]	≤ 5
*C*_*7*_	> 0.8	(0.78, 0.8]	(0.75, 0.78]	(0.7, 0.75]	≤ 0.7
*C*_*8*_	> 10	(4, 10]	(1, 4]	(0, 1]	0

### Methods

#### EFAST-cloud model

The EFAST method is an enhanced global sensitivity analysis technique that combines the Sobol method with the FAST method. The cloud model theory employs the concepts of expectation (*E*_*x*_), flexibility (*E*_*n*_), and hyper-entropy (*H*_*e*_) to address the issue of ambiguity in bridging qualitative concepts and quantitative descriptions^19^. In order to evaluate the development level of each subsystem of the CPUA, the EFAST method is used to determine the weight indicators and combines it with the Cloud model to improve the calculation of the traditional evaluation index. The following are the implementation steps^1,2^:

1) Determine the digital properties (*E*_*x*_, *E*_*n*_, *H*_*e*_) of each indicators, specifically the super-entropy. It is a constant that is determined through empirical observation and measurement. To mitigate the impact of system randomness on the findings, *H*_*e*_ is assigned a value of 0.01. The value of *E*_*x*_ is determined using the following mathematical formula^20^:

1 $$E_{x} = \frac{{C_{\max } + C_{\min } }}{2}$$

where *E*_x_ is expectation; *C*_*max*_ is the maximum value of the grade threshold; and *C*_*min*_ is the minimum value.

Cloud systems exhibit a shift from one grade to another when the evaluation grade nears the threshold due to their intrinsic properties. As a result, it exhibits ambiguity and should be classified into two classes simultaneously^21^:

2 $$\exp \left( { - \frac{{C_{\max } - C_{\min } }}{{9E_{n}^{2} }}} \right) \approx 0.5$$

Namely:

3 $$E_{n} = \frac{{C_{\max } - C_{\min } }}{2.355}$$

2) Determine the membership degree of each indicator that corresponds to a distinct grade, the positive cloud generator is employed. The following is the formula:

4 $$\mu = e^{{\frac{{ - \left( {x - E_{x} } \right)^{2} }}{{2E_{ni}^{2} }}}}$$

where* µ* is membership degree; *x* is index value; and *E*_*x*_ is the same as above.

3) Calculate the weight Wi of each indicator based on the data and the EFAST approach. To mitigate the effects of both positive and negative indicators, dimensionless treatment is conducted using the deviation standardization approach, bringing the result between 0 and 1^22^, then *C*_min_ = 0, *C*_max_ = 1.

Positive indicators:

5 $$x_{ij} = \frac{{x_{ij} - x_{ij}^{\min } }}{{x_{ij}^{\max } - x_{ij}^{\min } }}$$

Negative indicators:

6 $$x_{ij} = \frac{{x_{ij}^{\max } - x_{ij} }}{{x_{ij}^{\max } - x_{ij}^{\min } }}$$

where *x*_ij_ is the *j* index value of the year *i, x*_ij_^min^、*x*_ij_^max^ are the minimum and maximum values of this index.

Simlab 2.2, a mainstream software for uncertainty and sensitivity analysis, is utilized in this study. For the EFAST method, the sampling frequency is set to 65 times the number of input parameters, with a fixed total sample size of 12,000.

4) Determine the overall membership degree. The evaluation level is calculated based on the notion of maximal membership of the cloud model.

7 $$C_{mi} { = }\sum\limits_{i}^{n} {\mu_{mi} w_{i} }$$

where *μ*_*mi*_ and *C*_*mi*_ represent the membership and degree respectively when the *i*-th index evaluation grade is *m*.

Furthermore, to assess the robustness of the framework, a sensitivity analysis was performed on the key parameters. The weighting factors and threshold values were varied by ± 20% from their baseline settings, and the overall model behavior remained stable throughout these tests. This indicates that the results are not overly sensitive to specific parameter choices, thereby enhancing confidence in the reliability of the findings.

#### The improved coupling coordination degree model (ICCDM)

The CCD model is a robust analytical framework for quantifying both coupling states between interdependent systems and synergistic interactions among system variables. It is composed of a coupling degree (*C*) and a comprehensive development level (*T*), which can comprehensively evaluate the coordinated development degree among regional water, economy, and ecology systems.Structurally, both *C* and *T* derive from a unified comprehensive evaluation index (*Y*), the traditional comprehensive is commonly calculated by a general formula, as follows^23,24^:

8 $$Y_{1j} = \sum\limits_{i = 1}^{n} {\omega_{1} x_{1j} } ;\;Y_{2j} = \sum\limits_{i = 2}^{n} {\omega_{2} x_{2j} } ;\;Y_{3j} = \sum\limits_{i = 3}^{n} {\omega_{3} x_{3j} }$$

where, *Y*_1*j*_, *Y*_2*j*_, and *Y*_3*j*_ are the comprehensive evaluation indexes of each system; *w*_*1*_, *w*_*2*_, and *w*_*3*_ are the weight of each system indicator.

*Y* plays a very important role in measuring the degree of regional coupling; however, traditional evaluation methods suffer from issues such as insufficient accuracy and lack of comprehensiveness. Therefore, this study introduces the ECM as the evaluation model, thus making the evaluation results more accurate. This conversion aggregates the membership degrees across all evaluation grades (m = 1 to 5) into a single composite score, ensuring the result is bounded between 0 and 1. The calculation formula is as follows:

9 $$c_{i} = \frac{Cmi}{{\sum\limits_{i}^{n} {Cmi} }}$$

10 $$Y^{\prime} = c_{1} \times 1 + c_{2} \times 0.8 + c_{3} \times 0.6 + c_{4} \times 0.4 + c_{5} \times 0.2$$

where, ci denotes the proportion of comprehensive affiliation when the evaluation level is m. Y' denotes the CDI to improve the coupling coordination, and the larger the value, the better the development of the system.

Based on the above, the coupling degree (*C*), comprehensive development index (*T*), and CCD (*D*) are calculated as follows:

11 $$C_{j} = \left[ {\frac{{Y_{1j} \times Y_{2j} \times Y_{3j} }}{{\left( {\frac{{Y_{1j} + Y_{2j} + Y_{3j} }}{3}} \right)^{3} }}} \right]^{\frac{1}{3}}$$

12 $$T = \alpha Y_{1j} + \beta Y_{2j} + \gamma Y_{3j}$$

13 $$D = \sqrt {C \times T}$$

where, *C*_*j*_ is the coupling degree of each system in the *j*th year; *T*_*j*_ is the CDI in the *j*th year, and $\alpha ,\beta ,\gamma$ are the coefficients to be determined, $\alpha { + }\beta { + }\gamma { = }1$, due to the interaction between water, economy, and ecology, and therefore equally important, so $\alpha { = }\beta { = }\gamma { = 1/3}$; *D*_*j*_ is the CCD of the *j*th year, which can reflect the integrated level of the system as well as the coordinated state of the system’s development, $0 \le D_{j} \le 1$, and the smaller the *D*_*j*_ is, it means that the coordination status between the systems is poorer, and the bigger the *D*_*j*_ is, the better the coordinated status will be.

#### Spatial relationship analysis model

##### Spatial autocorrelation model

1. Global spatial autocorrelation

The global spatial autocorrelation is judged from the overall macroscopic point of view to determine the degree of elemental aggregation, and the calculation results are expressed by the global Moran’s I, which is calculated by following formula^25^:

14 $$I = \frac{{\sum\limits_{i = 1}^{n} {(D_{i} - \overline{D} )\sum\limits_{j = 1}^{n} {W_{ij} (D_{j} - \overline{D} )} } }}{{S_{ij}^{2} \sum\limits_{i = 1}^{n} {\sum\limits_{j = 1}^{n} {W_{ij} } } }}$$

where, *I* is the global spatial autocorrelation coefficient, *n* is the number of spatial units, ${S}_{ij}^{2}$ is the variance of the CCD of each city; *D*_*i*_ and *D*_*j*_ are the CCD of spatial units *i*, *j*, respectively; *W*_*ij*_ is the spatial weight of efficient matrix; and $\overline{D }$ is the average value of the CCD. The range of Moran’s I is [−1, 1]: when the *I* < 0, it indicates that the CCD of the WEE system is spatially negatively correlated; when the *I* > 0, it indicates that the two regions are positively correlated; when the *I* = 0, there is no correlation between the two regions.

(2) Local spatial autocorrelation.

The local spatial autocorrelation measures whether the high and low values of the element distribution are clustered in the local space and is calculated as follows^25^:

15 $$I_{i} = \frac{{(Di - \overline{D} )}}{{S_{ij}^{2} }}\sum\limits_{j = 1}^{n} {W_{ij} (Dj - \overline{D} )}$$

where, *Ii* refers to the local spatial autocorrelation coefficient. If *I*_*i*_ > 0, there is a spatial distribution characteristic of high-high (H-H) or low-low (L-L) agglomeration between the two cities, and vice versa, there is a spatial characteristic of high-low (H–L) or low–high (L–H) agglomeration.

##### Gravity model

The Gravity model is a mathematical model to analyze the ability of spatial interaction, the model has been widely used in a variety of disciplines, relatively mature. This study uses the gravitational model to determine the value of the mutual gravitational force between Zhengzhou and other cities to analyze the degree of interaction between the cities, and is calculated as follows^9–11^:

16 $${F}_{ij}={k}_{ij}\frac{{Q}_{i}{Q}_{j}}{{D}_{ij}^{r}}$$

17 $${k}_{ij}=\frac{{Q}_{i}}{{Q}_{i}+{Q}_{j}}$$

where: *F*_*ij*_ represents the gravity force between city *i* and city *j*; *K*_*ij*_ is the modified gravitational constant; *Q*_*i*_ is the mass of city *i*; *Q*_*j*_ is the mass of city *j*; *D*_*ij*_ is the geographic distance between city *i* and *j*, which is expressed as the straight-line distance from the geographic COG of the city group; and *r* is the gravitational attenuation coefficient, which is generally taken as the value of 2.

##### COG migration model

In order to reflect the dynamic changes of the CCD of the WEE system in the CPUA area, the COG migration model is introduced to represent the dynamic evolution characteristics in the time dimension, and the COG change responds to the trajectory of the CCD of the WEE system and is calculated as follows^9–11^:

18 $$a = \frac{{\sum\limits_{i = 1}^{n} {z_{i} a_{i} } }}{{\sum\limits_{i = 1}^{n} {z_{i} } }}\;\;b = \frac{{\sum\limits_{i = 1}^{n} {z_{i} b_{i} } }}{{\sum\limits_{i = 1}^{n} {z_{i} } }}$$

19 $$D = \sqrt {\left( {b_{(t + 1)} - b_{t} } \right)^{2} + \left( {a_{(t + 1)} - a_{t} } \right)^{2} }$$

where *z*_*i*_ is the attribute value of the *i* th space unit (i.e. CCD); *a*_*i*_ and *b*_*i*_ are the latitude and longitude values; *a* and *b* are the barycentric coordinates of the property; *t* is the year.

## Results

### Index weight calculation

The EFAST method is used to calculate the weights of 25 indicators in three subsystems. The weight calculation of the EFAST method can reflect the sensitivity of the indicators, and the calculation performance is stable and efficient. However, this method only uses the objective value of the index data. Considering the complexity of the WEE system and the important role of human activities in it, the subjective analytic hierarchy process (AHP) is adopted to couple the WEE system and calculate the combined weight, which makes the result more reasonable. The results are shown in Table 3.

**Table 3:** Results of weighting calculation based on the EFAST method (%).

Criterion layer	Index layer	EFAST	AHP	Combined
*A*: Water	*A*_*1*_	22.33	27.33	25.66
	*A*_*2*_	17.15	8.05	12.20
	*A*_*3*_	4.94	1.461	2.79
	*A*_*4*_	10.94	22.294	16.22
	*A*_*5*_	13.94	11.253	13.01
	*A*_*6*_	5.45	4.015	4.86
	*A*_*7*_	4.35	2.328	3.30
	*A*_*8*_	7.51	2.97	4.91
	*A*_*9*_	7.81	13.625	10.71
	*A*_*10*_	5.58	6.674	6.34
*B*: Economy	*B*_*1*_	13.39	30.739	22.34
	*B*_*2*_	10.38	12.603	12.59
	*B*_*3*_	11.55	27.515	19.62
	*B*_*4*_	13.97	14.904	15.89
	*B*_*5*_	12.47	4.105	7.88
	*B*_*6*_	12.74	3.463	7.31
	*B*_*7*_	25.51	6.672	14.36
*C*: Ecology	*C*_*1*_	20.24	15.026	19.75
	*C*_*2*_	8.29	28.703	17.47
	*C*_*3*_	19.61	7.908	14.10
	*C*_*4*_	4.74	4.027	4.95
	*C*_*5*_	21.25	3.735	10.09
	*C*_*6*_	7.33	2.191	4.54
	*C*_*7*_	10.62	11.785	12.67
	*C*_*8*_	7.92	26.625	16.44

From the combined weights of indicators in Table 3, the indicators with the highest weights in the three systems are *A*_*1*_ (Per-capita water resource), *B*_*1*_ (Per-capita GDP), and *C*_*1*_ (Greening coverage in built-up areas), respectively, which means that these three indicators play a key role in the evaluation of each system. On the contrary, *A*_*3*_ (Water production coefficient), *B*_*6*_ (Percentage of investment in water supply and drainage), and *C*_*6*_ (Drainage pipe density) are the least weighted indicators in the three systems, respectively, and have a weaker impact.

### The development level of the WEE system

#### Three subsystems

**Fig. 2:**
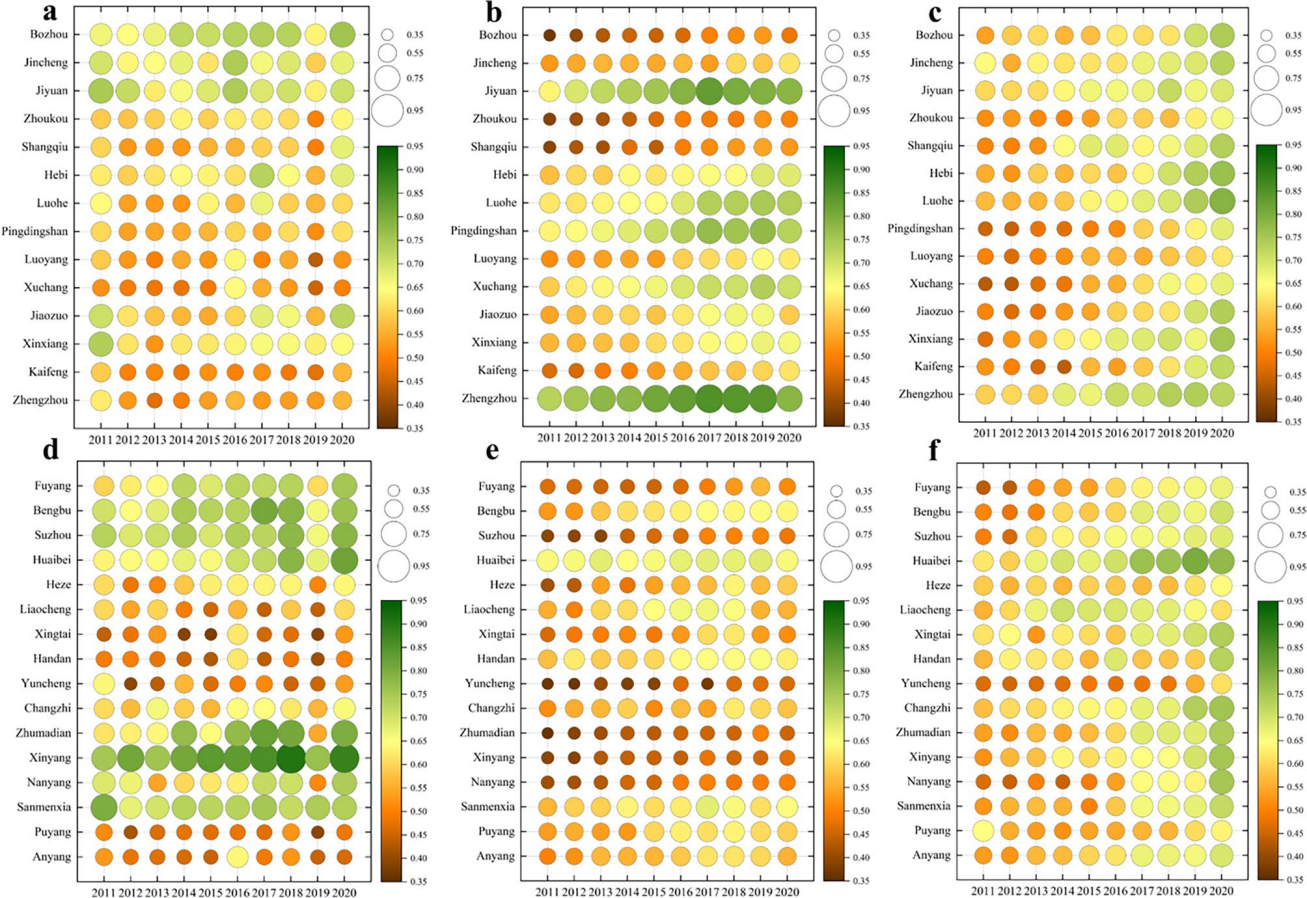
CDIs of three subsystems in the CPUA: (**a**) Water system in the CDA, (**b**) Economy system in the CDA, (**c**) Ecology system in the CDA, (**d**) Water system in the NCDA, (**e**) Economy system in the NCDA, (**f**) Ecology system in the NCDA.

The CDIs of each urban water, economy, and ecology system were calculated according to the ECM. In order to understand the changes in different regions more clearly, the bubble diagrams shown in Fig. 2 were drawn. As can be seen from the figure, the CDIs of three subsystems in various cities of the CPUA have great inter-annual changes, showing a general trend of fluctuation and rise.

By calculating the standard deviation of the CDIs of 30 cities in 10 years, the fluctuation degree of the water system in each city can be obtained. The three cities with the smallest standard deviation are Sanmenxia, Jiaozuo, and Kaifeng. The inter-annual CDI changes of these cities are stable, and they are usually in areas with extreme abundance or shortage of water supply, and are less influenced by natural variation. The three cities with the largest standard deviation are Zhumadian, Nanyang, and Xingtai, which are highly susceptible to the influence of natural conditions due to the drastic changes in the inter-annual composite index. Through engineering means and government regulation measures, the situation of uneven distribution of regional water resources can be alleviated, the inherent deficiency can be made up, and the sustainable development of the water system can be guaranteed.

By calculating the 10-year extreme deviation of the CDI of the economy system in the CPUA, the magnitude of change in each city during the study period can be obtained. The three cities with the largest extreme deviation are Heze, Jiyuan, and Liaocheng, indicating that these cities have obvious inter-annual changes in the CDI, with a clear tendency to improve. However, the economic development level of some of the cities in this category is not outstanding, which is mainly due to the irrationality of the original economic structure of these cities. Through the adjustment of policies and the exploration of regional development modes in recent years, their industrial structure has been gradually optimized, which has effectively improved their economic and social development level. The three cities with the smallest extreme difference are Huaibei, Handan, and Jincheng, which have insignificant changes in the CDI between years, and their common point is that their economic development is in a good condition, but due to the slow process of development, which results in the limitation of their economic and social development level. Therefore, this group of cities should fundamentally find out the factors restricting their development, fully utilize their resources and original industrial structure, and further enhance their development level.

By calculating the CDI gap of the ecology system between the beginning and the end of the study period, the variations of each city can be determined. The three cities with the biggest changes are Nanyang, Luoyang, and Hebi, which have a slow urbanization process, a lack of environmental protection awareness, and incomplete supporting facilities at the initial stage of the study period. With the concept of ecological civilization and the introduction of policies, the awareness of ecological environmental protection in each region has been strengthened. The three cities with the smallest changes are Puyang, Liaocheng, and Heze, among which Puyang even has a negative growth. The common characteristic of these cities is that with the rapid economic and social development, they failed to balance the relationship between the economy and ecology, and increased the pressure on the environment without appropriate environmental compensation. As a result, the ecological indicators of such cities have not improved significantly and have even deteriorated to some extent.

#### The WEE composite system

##### Temporal evolution

**Fig. 3:**
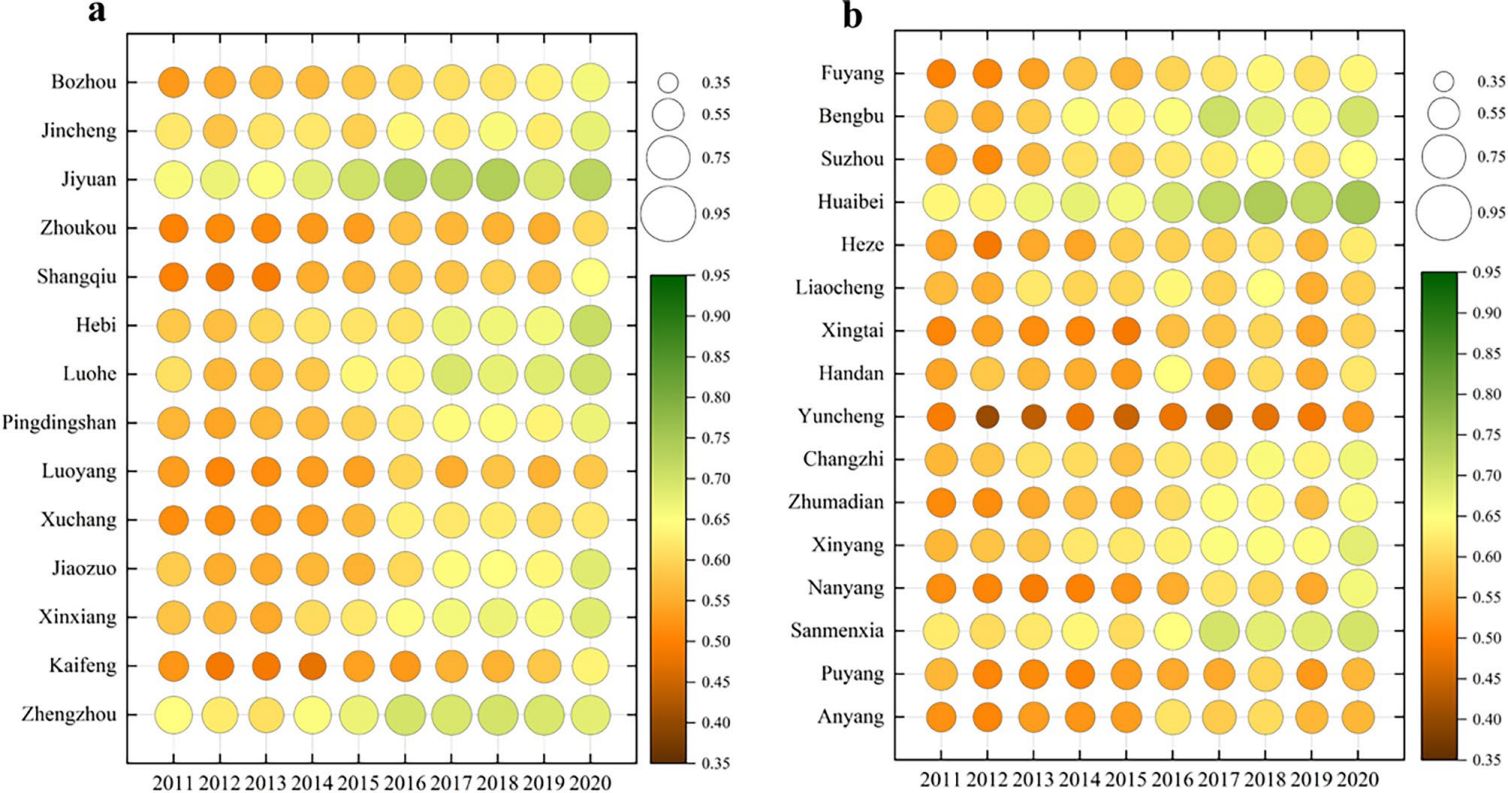
CDI of WEE coupled system in the CPUA: (**a**) urban conditions in CDA; (**b**) urban conditions in NCDA.

In order to analyze the temporal evolution of the development level of the WEE composite system, the CDI of the target level was used to draw Fig. 3. As can be seen from the figure, although the change amplitude of the CDI of the WEE coupled system in various cities varies greatly during 2011–2020, overall they all show a positive trend year by year.

The magnitude of the enhancement can be derived by calculating the CDI difference of the WEE composite system between 2011 and 2020 for the 30 cities. The three cities with the largest enhancement are Nanyang, Zhumadian, and Shangqiu, and the common characteristic of these cities is that their target layer’s CDI is low at the start, but with the acceleration of the development process of the CPUA, the economy of these cities has developed rapidly, and the environment has improved significantly, which has effectively contributed to the benign evolution of the target layer. The three cities with the smallest enhancement in the target layer’s CDI are Puyang, Liaocheng, and Zhengzhou. The target level CDI of these cities shows a polarized situation, with Zhengzhou as the representative city with a relatively good initial development level, so compared to other cities, there is limited room for improvement. As for Puyang and other cities, their initial development level is poor, but the development mode is relatively backward, so the overall development level is not excellent, and it is necessary to find a suitable development mode, learn from the development experience of advanced regions, and seek a breakthrough.

##### Spatial evolution

**Fig. 4:**
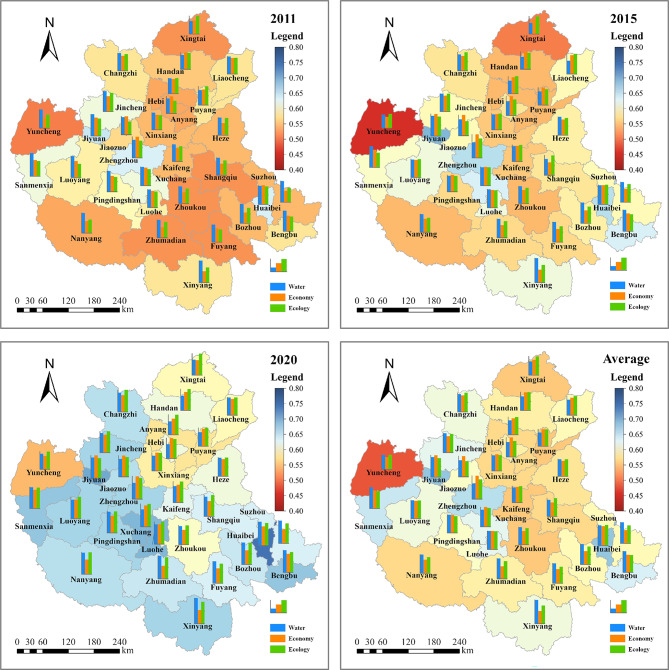
Spatial distribution of the CDI for the target layer (created using ArcGIS 10.8, https://www.esri.com, based on the standard map GS(2019)1822).

In order to analyze the spatial evolution of the development level of the WEE composite system, the data from 2011, 2015, 2020, and the average value are selected to draw a spatial distribution map as shown in Fig. 4. The CDI of the WEE target layer in the CPUA has all been improved to different degrees. Overall, the CDA is in better shape than the NCDA.

By analyzing the three subsystems of each city, it is found that the development level of their subsystems varies greatly, and they can be broadly classified as “resource-endowed” “economic-developed” and “environment-friendly”. Among them, the typical resource-endowed cities are Sanmenxia and Bengbu, whose water resources have a significant advantage over other cities. Although their economic development is relatively general, their resource carrying capacity is better, and they can enhance the level of other systems through rational development. Typical cities of economic-developed are Zhengzhou and Jiyuan. Although their water resources are general, their economic advantages are outstanding, and their industrial structure is reasonable, so they can fully utilize their limited resources to improve the overall level. Typical environment-friendly cities are Huaibei and Changzhi, which have better urban greening, better water treatment facilities, and smaller industrial wastewater discharges, and are able to obtain higher economic benefits with smaller environmental costs.

### Coupled coordination analysis

#### Coupling coordination evaluation of the dual system

To further determine the CCD between the systems, this study adopts the ICCDM to measure the dual system coupling coordination, and analyzes it from the perspective of the urban agglomeration as a whole and the multi-year average situation of each city.

#####  “Water-economy” system

**Fig.5:**
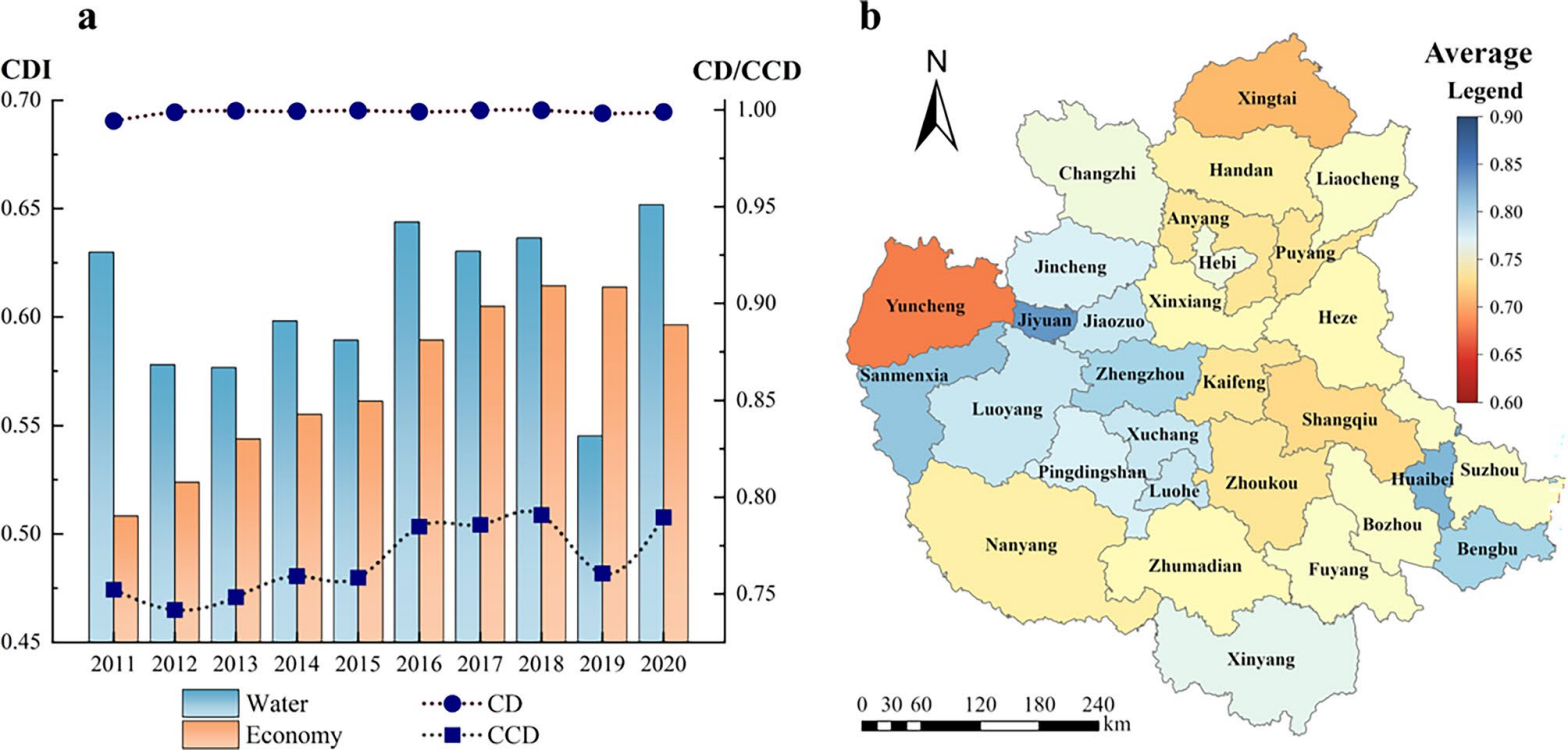
Coupling coordination situation of “water-economy” dual system in CPUA: (**a**) variation trend of CD and CCD; (**b**) multi-year average CCD (created using ArcGIS 10.8, https://www.esri.com, based on the standard map GS(2019)1822).

Based on the CDI, CD, and CCD of the water and economy systems, a trend diagram is drawn as shown in Fig. 5(a). It shows that the CD of the “water-economy” dual system of the CPUA is high, with an average value of 0.9988, but it fluctuates drastically, with significant inter-annual differences. Its CCD is in the range of 0.74–0.80, presenting a general trend of improvement, although the magnitude of change is relatively small. With the exception of 2019, in the “water-economy” dual system of the CPUA, the water system plays a leading role, but with the rapid development of the economy, the gap between the two systems has gradually decreased.

The CCD of the coupled “water-economy” system of the cities in the CPUA during the period of 2011–2020 is shown in Fig. 5. The CCD of the coupled “water-economy” system of each city varies significantly. From the figure, it can be seen that on the spatial scale, the western part is better than the eastern part, and the CDA is better than the NCDA in the long term. The main reason is that the CDA has a relatively good water resource management and allocation mechanism that ensures the effective use and reasonable distribution of water resources, so as to meet the needs of economic development. Moreover, the industrial structure of the CDA is more reasonable, and the enterprises with high water consumption and high pollution are gradually replaced by high-tech industries, forming an industrial pattern of green economy, which helps to reduce excessive consumption, thus improving the CCD of the “water-economy” system.

The five cities with the largest multi-year averages are categorized as Jiyuan, Huaibei, Sanmenxia, Bengbu, and Zhengzhou, and the CCD is all greater than 0.8. Such cities exist at a high level of excellence in one system, it is important to further balance the relationship between the two systems, give full play to the kinetic energy of the advantageous system, and guarantee the balanced development of the region. The five cities with the smallest average value of CCD are Yuncheng, Xingtai, Shangqiu, Puyang, and Kaifeng, which generally have backward economic development and poor water resource development and utilization.

#####  “Water-ecology” system

**Fig.6:**
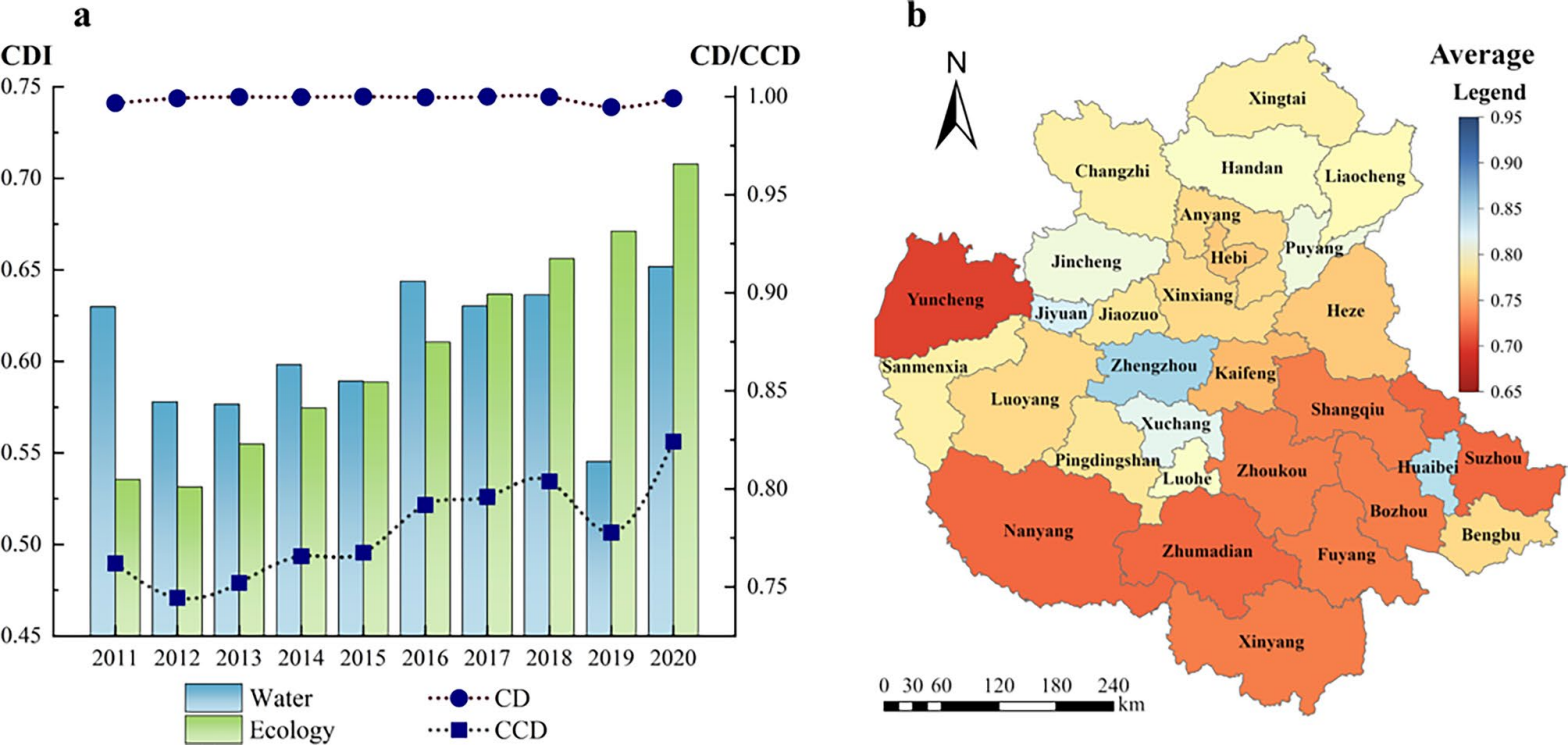
Coupling coordination situation of “water-ecology” dual system in CPUA: (**a**) variation trend of CD and CCD; (**b**) multi-year average CCD (created using ArcGIS 10.8, https://www.esri.com, based on the standard map GS(2019)1822).

The CD and CCD of the “water-ecology” system of the CPUA from 2011 to 2020 are shown in Fig. 6. It shows that the CD of the CPUA is in the range of 0.9946–1, with an average value of 0.9989, which is relatively stable, and the CCD is in the range of 0.74–0.83, showing a trend of improvement year by year. Among them, 2019 was affected by an extreme drought, which made the CDI of the water system drop sharply, thus leading to the reduction of both CD and CCD in that year as well. With the enhancement of the whole society’s awareness of ecological environment protection, the situation of the ecology system has been greatly improved, therefore the dominant role in the “water-ecology” dual system has gradually changed from the water system to the ecology system.

From Fig. 6, we can see that the CCD of NCDA is better than that of CDA. The main reason is that the CDA is densely populated and industrially developed, which has a greater demand for water resources, and the natural environment is under some pressure due to the advanced level of economic growth. Although the overall situation of NCDA is better, the fluctuation of its CCD is more drastic, and it is more sensitive to the interference of external factors. The CCD of the NCDA shows a pattern of east > south > west > north. The reason why the eastern demonstration area for undertaking industrial transfer is better than the other areas is mainly due to the fact that it has constructed a modernized industrial system through the adjustment of its development, reduced pollution, and improved the quality of the ecological environment. In addition, the CCD of the water-ecology system varies significantly among cities. In terms of the average value, the five cities with the best CCD status are Xinyang, Huaibei, Jiyuan, Suzhou, and Sanmenxia; the five cities with the worst CCD status are Yuncheng, Puyang, Hebi, Kaifeng, and Xinxiang.

#####  “Economy-ecology” system

**Fig.7:**
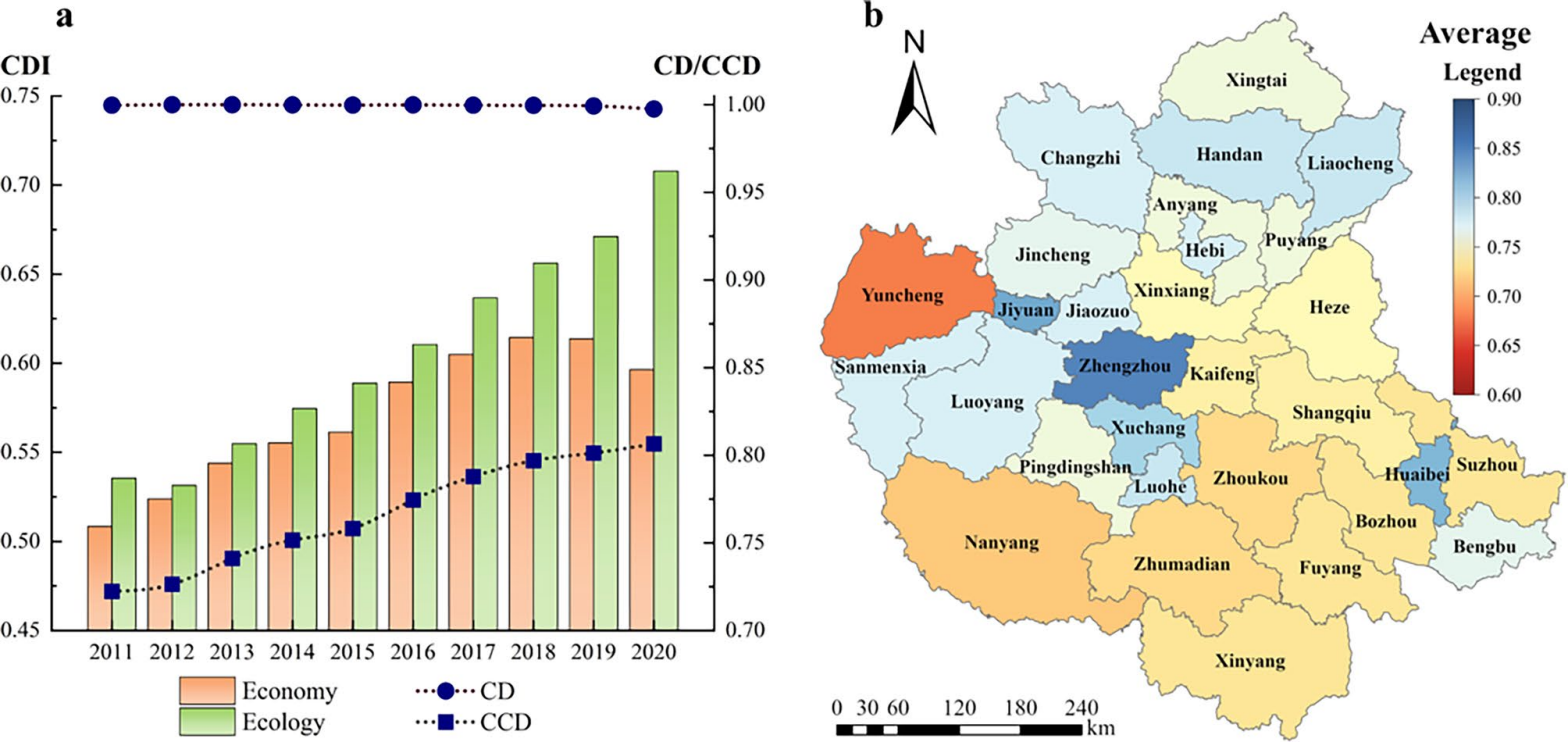
Coupling coordination situation of “economy-ecology” dual system in CPUA: (**a**) variation trend of CD and CCD; (**b**) multi-year average CCD (created using ArcGIS 10.8, https://www.esri.com, based on the standard map GS(2019)1822).

During the period of 2011–2020, the evaluation results of CD and CCD of CPUA’s “economy-ecology” system are shown in Fig. 7. From the figure, the CD of the two systems is high, with an average value of 0.9954, and basically shows an increasing trend, while the CCD shows an increasing trend year by year, with a CCD between 0.7223 and 0.8061. The minimum and maximum values of the CCD appear in 2011 and 2020, respectively. In addition, the ecology system has always been in the dominant position in many years, which also shows that with the development of ecological civilization construction, the ecology system has been strongly guaranteed.

The multi-year average CCD for the economy-ecology dual system in CPUA is shown in Fig. 7. From the figure, the CCD of CDA is significantly higher than that of NCDA. CDA has a higher level of economic development, intensive economic activities, a more optimized industrial structure, and stronger economic strength and competitiveness, thus having more resources and funds for ecological environmental protection and governance. At the same time, the CDA has a higher degree of urbanization, a concentrated population, and a advanced urban infrastructure, with stronger urban service functions and radiation effects.

There is a difference in the level of coordinated development of the “economy-ecology” system in each city, and the five cities with the best status are Zhengzhou, Jiyuan, Huaibei, Xuchang, and Luohe. The five cities with the worst coordination are Yuncheng, Nanyang, Zhoukou, Zhumadian, and Fuyang. The level of coordination is relatively high in the neighboring cities due to the radiation effect of Zhengzhou, the center city. Therefore, the cities with better economic conditions should emphasize their market vitality to drive the economic development of their neighboring cities, thus improving the coordination of the whole system. Besides, the gap between the coordinated development levels of cities is decreasing year by year, and the coordinated development of the “economy-ecology” system of the CPUA tends to be balanced and stable.

##### Analysis of coupled coordinated development patterns

**Fig. 8:**
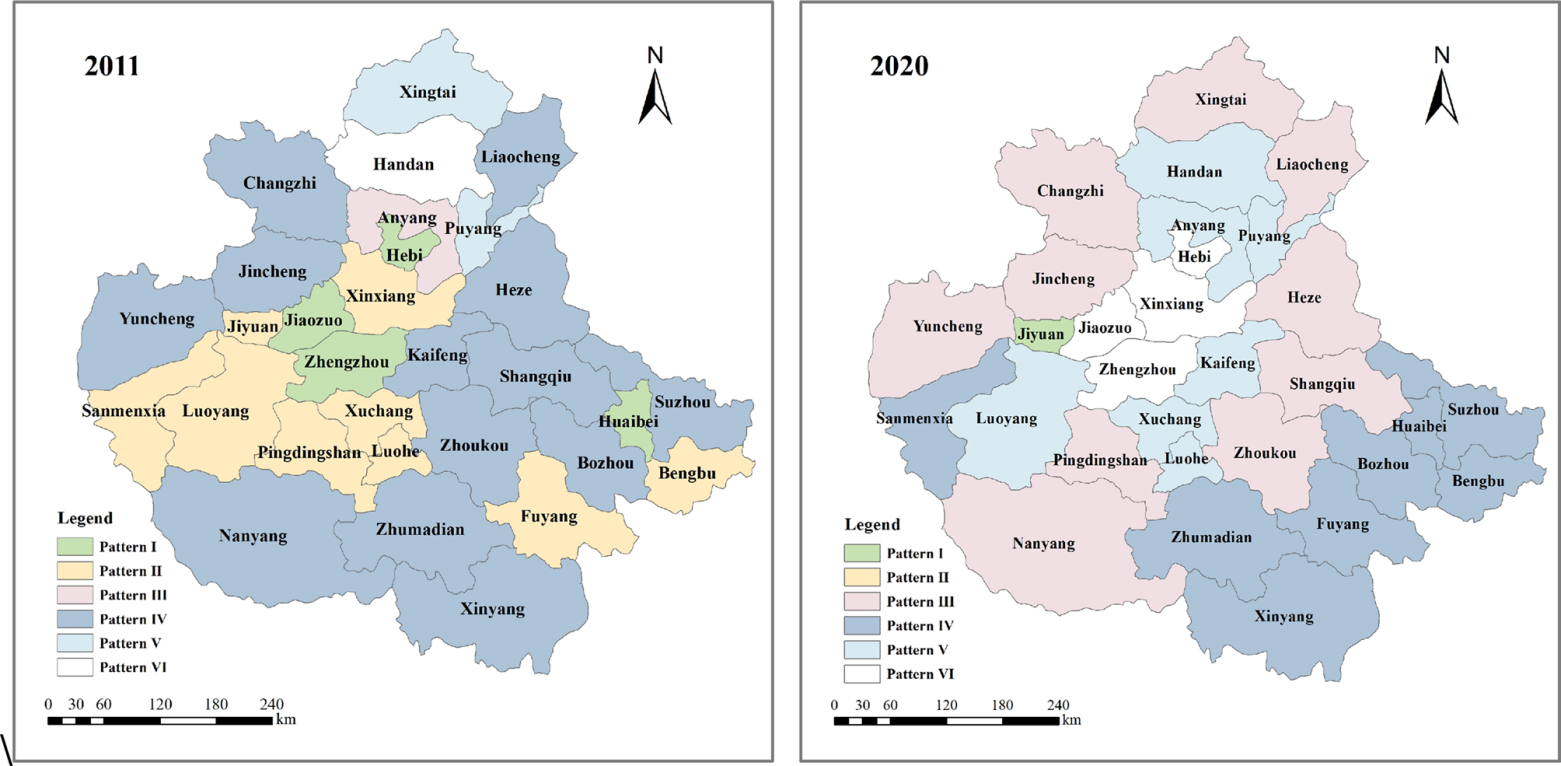
Spatial distribution of dual-system development patterns in the CPUA (created using ArcGIS 10.8, https://www.esri.com, based on the standard map GS(2019)1822).

In order to further clarify the constraints and development paths of the coordinated relationship of the WEE system of the cities in the CPUA, this study compares the CCD of the composite dual systems, and the one with the larger CCD is named the advanced development dual system, which indicates that the two subsystems are relatively well-developed. On the contrary, the dual system with the smaller CCD was recorded as the lagging development dual system, indicating that the two subsystems are in an unbalanced state and relatively poorly developed. Based on this, six different developmental patterns were set, as shown in Table 4. And the starting two years of the study period, i.e., 2011 and 2020, were selected as representative years, and the distribution map of municipal scale development patterns in the CPUA as shown in Fig. 8 was drawn.

**Table 4:** CPUA coupled coordinated development patterns based on numerical classification. (Advanced: the dual system with the higher CCD; Lagging: the dual system with the lower CCD).

Pattern	Advanced	Lagging
I	Water-economy	Water-ecology
II	Water-economy	Economy-ecology
III	Water-ecology	Water-economy
IV	Water-ecology	Economy-ecology
V	Economy-ecology	Water-economy
VI	Economy-ecology	Water-ecology

As can be seen from the Fig. 8, the pattern of the dual system of the CPUA has changed significantly. Among them, the number of pattern I cities has changed from 4 in 2011 to 1 in 2020, and the number of pattern II cities has even changed from 9 to 0. The common characteristic of these two models is that the best CCD is the “Water-Economy” dual system, with a poor ecological level. On the other hand, the number of pattern III cities increased from 1 to 10, and this model is characterized by the best CCD of the “Water-Economy” dual system, with a more ideal ecological level. The above two significant changes show that the awareness of environmental protection in the CPUA has been significantly increased at this period, and the ecological environment has been improved. In addition, in 2020, the CDA-ZZMA all become pattern V and pattern VI, both models use the “Economy-Ecology” dual system as the advanced development dual system, which characterizes their rapid economic development as core regions of urban agglomerations.

#### Coupling coordination evaluation of the WEE composite system

After evaluating and analyzing the coupling and coordination status of the two subsystems, this study evaluates the coupling coordination and analyzes the temporal and spatial evolution of the WEE composite system at the urban agglomeration scale, urban partition scale, and municipal scale, respectively.

##### Urban agglomeration scale

**Fig. 9:**
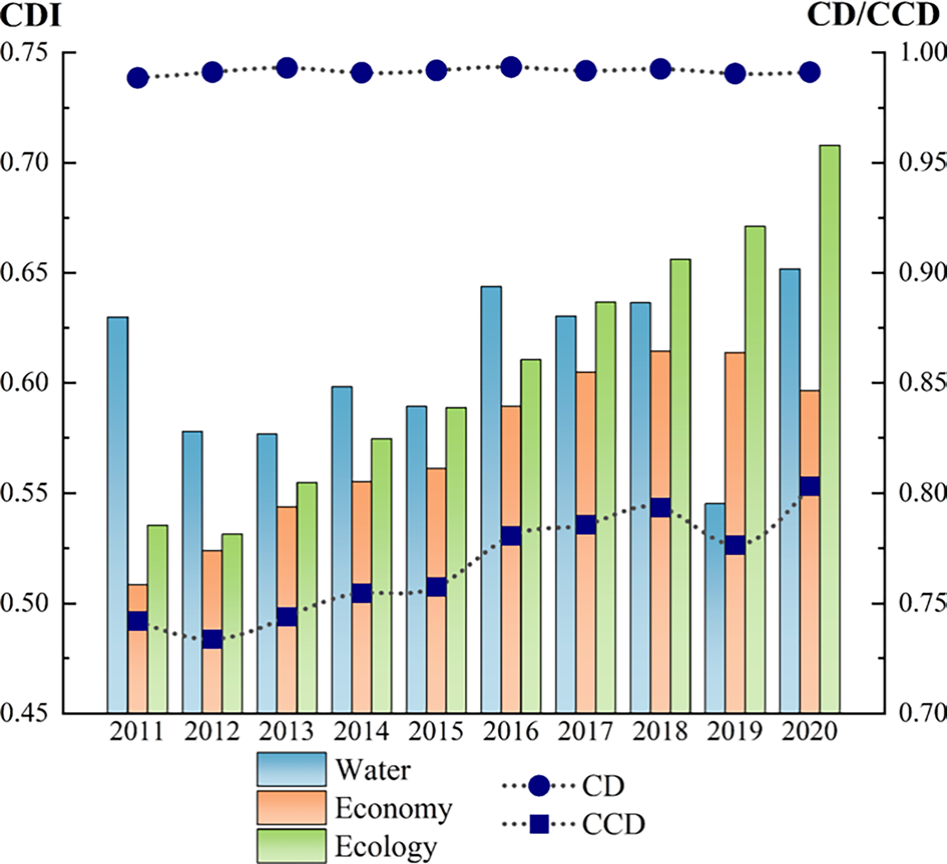
Trend of CD and CCD of WEE system in the CPUA.

In order to reflect the coupling coordination development of the WEE composite system in the CPUA, the CD and CCD changes are shown in Fig. 9. From the figure, it can be seen that the CD of the WEE composite system in the CPUA is always at a high level, fluctuating between 0.98 and 1.0, with an average value of 0.9915. The CCD as a whole shows an improving trend, with the minimum and maximum values of the CCD of 0.7337 in 2012 and 0.8033 in 2020, and an average annual growth rate of 0.90%.

During the period 2011–2020, the CD maximum and CCD maximum of the WEE system in the CPUA appeared at different times, mainly due to the fact that CD is used to describe the degree of interaction between the systems, whereas CCD considers the degree of synergism between the internal components on the basis of the degree of coupling, and what is emphasized is the overall effect of synergism between the internal modules of the system. For example, in 2011, although the CD was lower, the water system was able to compensate for the shortcomings of the economy and ecology systems due to its extremely high level of CDI, which improved the CCD to a certain extent, whereas in 2012, although the gap between the CDI of the three subsystems decreased, the development was all at a low level, which resulted in a CCD status is unsatisfactory. In addition, during the period of 2011–2020, the dominant position of the three systems changed from the water system to the ecology system, indicating that with the deepening of the concept of ecological civilization and the government’s emphasis on ecological protection, the ecology system of the CPUA has significantly improved.

##### Urban partition scale

**Fig. 10:**
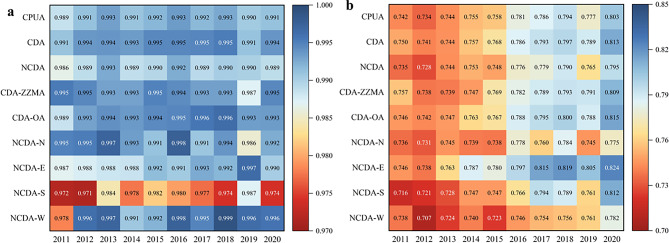
The CD and CCD changes of the WEE system in urban partition scale: (**a**) CD, (**b**) CCD.

The CPUA contains many cities, and the characteristics and development status of cities in different areas vary. In order to better analyze the situation within the urban agglomeration, this study calculates the CD and CCD of different urban partitions (Fig. 10).

From Fig. 10(a), it can be seen that there are some differences in the CD between the partitions of CPUA, and the CD of the CDA is always better than that of the NCDA, with an average value of 0.9936 and 0.9897, respectively, and the change trend of the CDA is more stable. For the CDA, there is a big difference between the CD trend of CDA-ZZMA and other regions, but the CD of CDA-ZZMA shows a decreasing trend, and the CD of other regions shows an increasing trend. The CDA-ZZMA is located in the center of Henan Province, with convenient transportation resources and strong economic strength, which helps it achieve better results in economic, social, and ecological development. However, the challenges of industrial restructuring and increasing pressure on resources and the environment have led to a downward trend in its coupling. For NCDA, the CD shows a pattern of West > North > East > South. The NCDA-W promotes the synergistic development of industries and the optimization of economic structure in the region through innovation-driven and industrial transformation and upgrading. At the same time, the region’s water resource endowment is relatively good, and the efficient development of the economy can promote the optimization of the allocation of water resources in the region and the benign evolution of the ecological environment.

From Fig. 10(b), it can be seen that there are obvious differences in the CCD among urban partitions, and the CCD of CDA has always maintained a certain advantage over that of NCDA, with the average values of its CCD being 0.7737 and 0.7614, respectively, and the CCD of the CDA has less fluctuation and the development is more stable. In the CDA, the average values of the CCD of CDA-ZZMA and CDA-OA are 0.7713 and 0.7751, respectively, and the CCD of CDA-OA is better than that of CDA-ZZMA in most cases, with the exceptions of 2011, 2015, and 2019. The main reason is that the CDA faced water shortage and severe drought in these three years, and the CCD of other regions was disturbed by the lack of natural endowment of water resources because of their strong dependence on natural resources. The CDA-ZZMA, on the other hand, has a well-developed industrial structure and water supply and demand structure, thus there is some resistance to fluctuations in natural endowments. Among the NCDA, the gap in the CCD of each region is relatively small, with values around 0.75, showing the overall situation of East > South > North > West. The NCDA-E has a superior geographic location, which is a hub connecting North, Central, and East China with convenient transportation. The superior geographic location is conducive to the allocation and efficient use of resources, and the region’s water resources are in good condition, which can support the development of economic society and the ecological environment.

##### Municipal scale

1. Temporal evolution

**Fig. 11:**
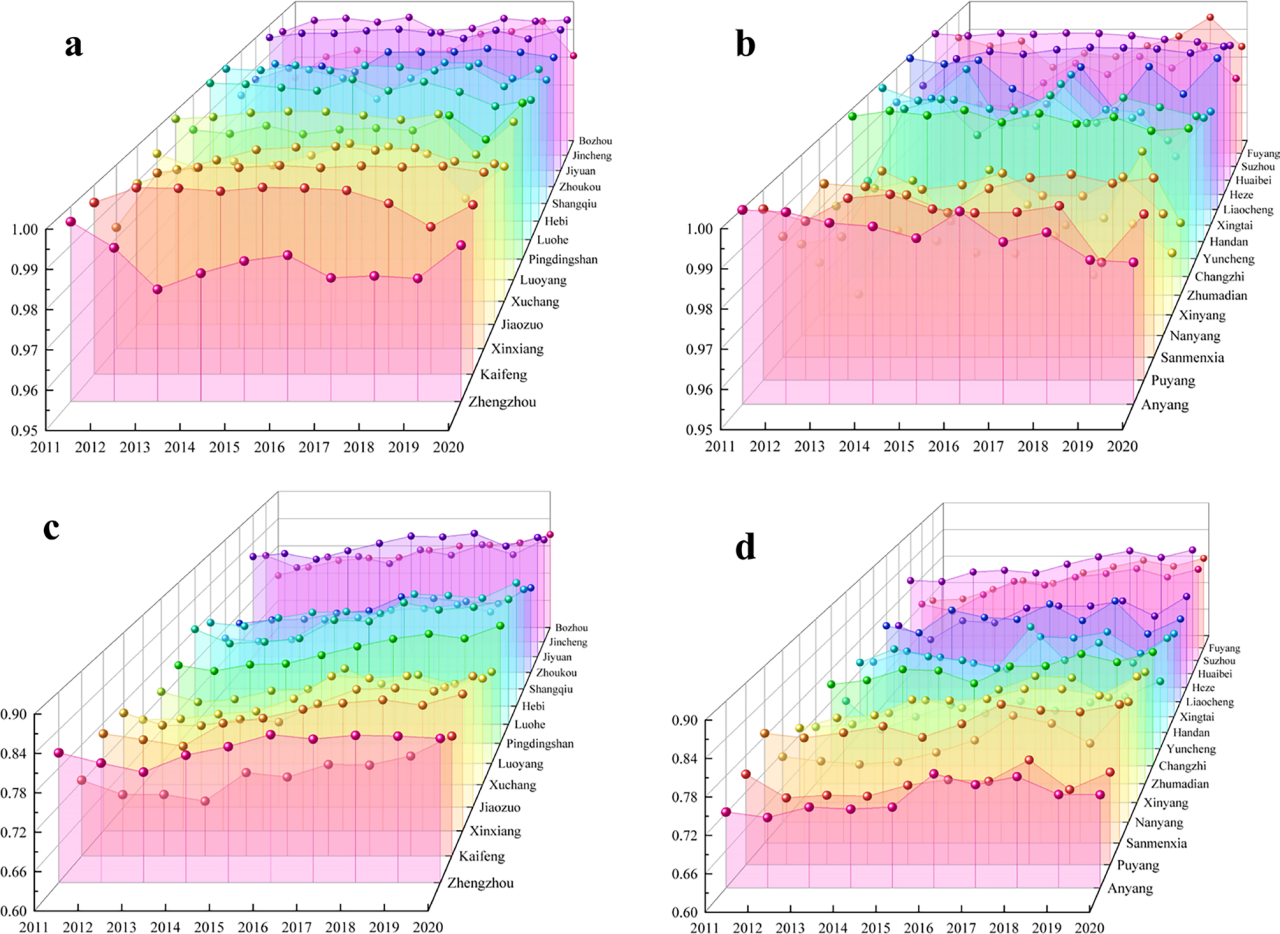
CD and CCD of WEE system in the CPUA: (**a**) CD conditions in CDA, (**b**) CD conditions in NCDA, (**c**) CCD conditions in CDA, (**d**) CCD conditions in NCDA.

In order to analyze the spatial evolution patterns of CD across cities in the CPUA, based on the results of CD calculation of the WEE system in each city, the waterfall diagrams as shown in Fig. 11(a),(b) were drawn. As can be seen from the figure, the CD changes of each city are relatively small, and the CD of nearly half of the cities shows a decreasing trend. Yuncheng, Sanmenxia, and Luoyang have the greatest degree of improvement, with an increase in CD of about 0.015, and Anyang, Handan, and Zhengzhou have the most obvious degree of deterioration. For the cities with significant CD improvements, the development gap between their water, economy, and ecology systems is getting smaller and smaller, but the overall growth rate is still to be adjusted. For cities with significant deterioration in CD, it is usually due to the sharp fluctuation in the status of the water system, and the economy and ecology systems show a high growth rate, which increases the difference between the composite indices of the three subsystems.

According to the CCD waterfall map of the WEE system in the CPUA drawn as Fig. 11(c),(d), the CCD of each city shows an increasing trend, and through the calculation of the difference between the CCD at the beginning and the end of the study period, it is concluded that the three cities with the largest increase in the CCD are Zhumadian, Shangqiu, and Nanyang, with an increase of about 0.1. The characteristic of such cities is that they are located in areas with abundant water resources, and the CDI of the water system is at a relatively high level. However, because they are still in the exploratory stage of economy system reform, their economic level is not outstanding, which leads to insufficient attention to environmental protection. However, with the relevant policy guidance and industrial upgrading, its economy has been significantly improved, and at the same time, it has increased the management of ecological environment, which has significantly improved the condition of ecology system, forming a pattern of low starting points and high growth rates, and thus making the increase in CCD remarkable. The three cities with the smallest increase in CCD are Puyang, Liaocheng, and Zhengzhou, with an increase of about 0.02. For this kind of city, they usually have excellent economic strength, but because of the greater demand for resources in their development, there is a competitive relationship between the systems, which leads to limited CCD.

(2) Spatial evolution

**Fig. 12:**
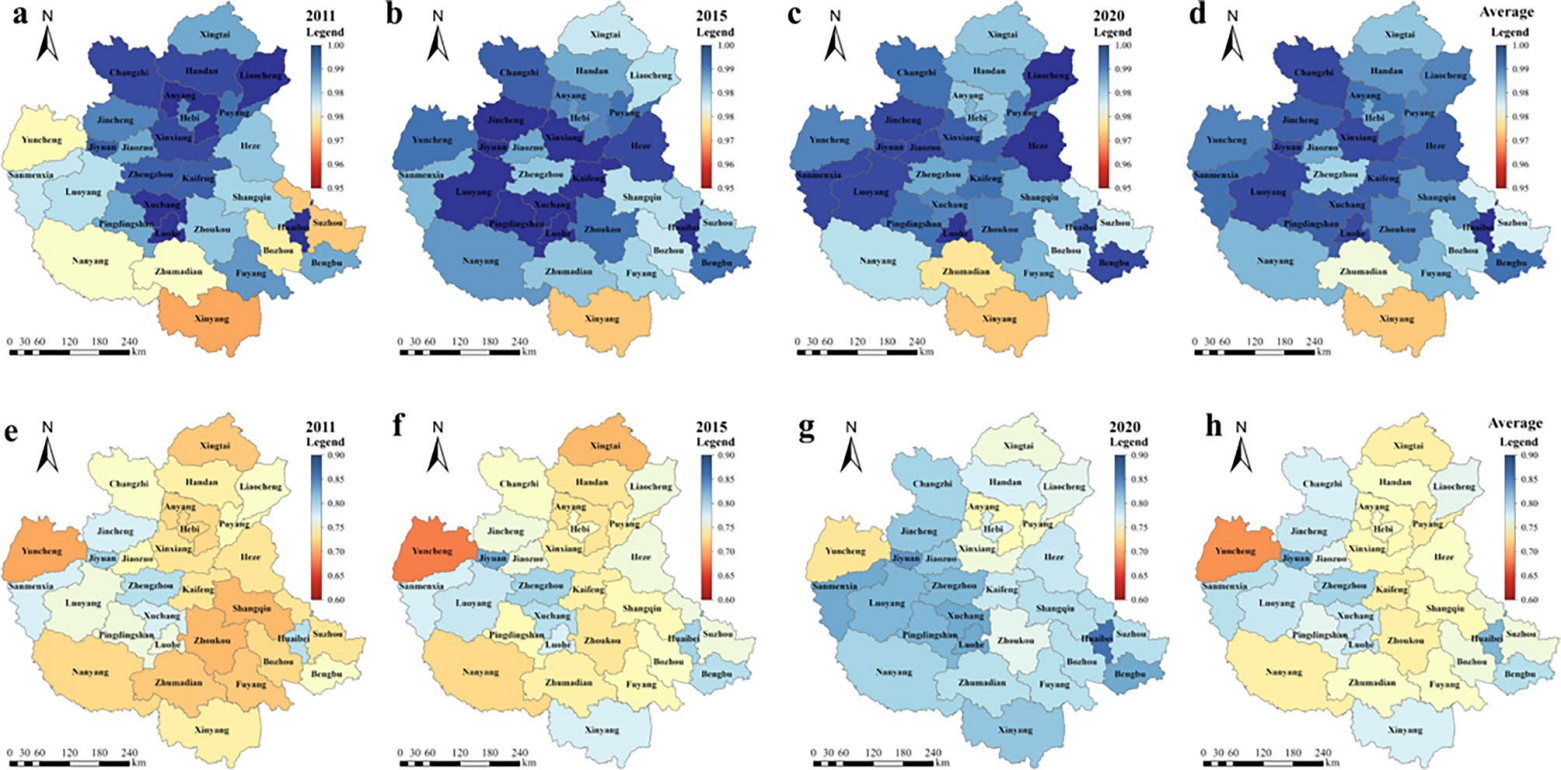
Spatial evolution in the CD and CCD of WEE system in the CPUA: (**a**) CD in 2010, (**b**) CD in 2015, (**c**) CD in 2020, (**d**) average CD over 10 years, (**e**) CCD in 2010, (**f**) CCD in 2015, (**g**) CCD in 2020, (**h**) average CCD over 10 years (created using ArcGIS 10.8, https://www.esri.com, based on the standard map GS(2019)1822).

In order to better understand the spatial evolution patterns of CD and CCD in different cities of the CPUA, 2011, 2015 and 2020 were selected as typical years, and GIS maps as shown in Fig. 12 were drawn. Meanwhile, the average values of CD and CCD from 2011 to 2020 were calculated and plotted, which were used to analyze the distribution pattern of the CPUA throughout the study period.

As can be seen from Fig. 12(a)–(d), the CD of the cities in the CPUA has shown a fluctuating trend, and is generally improving year by year. Among them, the CD in the southwest region is worse, and the center and north are better. The five cities with the best average are Huaibei, Luohe, Luoyang, Xinxiang, and Jiyuan, whose CD is above 0.995; the five cities with the worst average are Xinyang, Zhumadian, Suzhou, Bozhou, and Zhengzhou, whose CD is around 0.96. With the gradual development of the CPUA, the gap between regions is also gradually decreasing.

As can be seen in Fig. 12(e)–(h), the CCD of each city in the CPUA has improved significantly. Zhengzhou, as the core city of the urban agglomeration, has steadily maintained a high level over the years, while some cities in Shanxi and Hebei provinces have lagged behind. From the perspective of the average level of all years, the five cities with the best CCD status are Jiyuan, Huaibei, Zhengzhou, Sanmenxia, and Bengbu, with the average value of the CCD around 0.80. These cities can be categorized into two types, namely, the type of advantageous economic development represented by Zhengzhou and Jiyuan, and the type of advantageous water resources represented by Sanmenxia and Bengbu. Although the development of these three systems is not sufficiently balanced, the CCD is enhanced by the significant strengths of one system that compensate for the weaknesses of the others. The five cities with the worst conditions are Yuncheng, Kaifeng, Zhoukou, Xingtai and Puyang, with an average value of about 0.73, characterized by the fact that they are basically located in regions with poor water resource endowment and the overall development level is poor due to the backwardness of economic development. Such cities should actively explore the paths suitable for their development, and use the development of a certain sub-system as an entry point for the development of other subsystems, so as to form a coordinated and orderly development pattern.

### Spatial correlation analysis of CCD

#### Spatial autocorrelation analysis

##### Global spatial autocorrelation

**Fig. 13:**
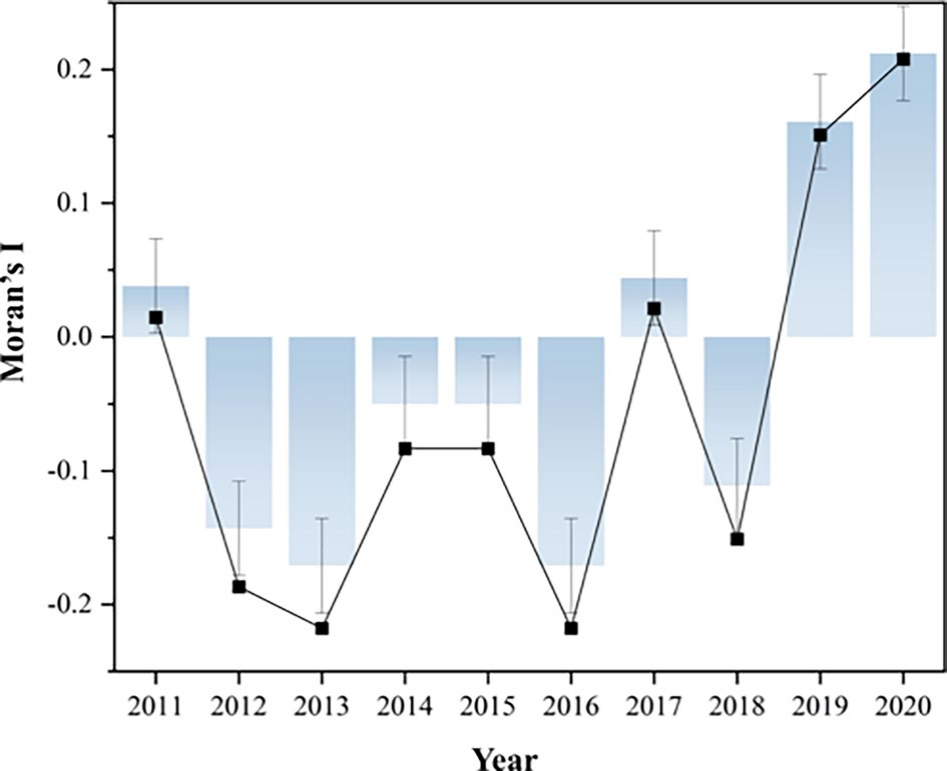
Trend of CCD Moran’s I of the WEE system in CPUA.

In this study, the univariate Moran’s I value of the CCD of the WEE system from 2011 to 2020 was calculated, and the Moran’s I change figure of the CCD in the CPUA shown in Fig. 13 was plotted.

As can be seen from Fig. 13, for the CCD of the WEE system in the CPUA, the univariate Moran’s I fluctuates violently, of which there are four positive values (i.e., positive correlation) in 2011, 2017, 2019, and 2020, respectively, and the Moran’s I of the remaining six years is negative, and all of them pass the significance level test, which means the existence of more obvious spatial correlation and spatial dependence of the CCD of urban agglomeration. In addition, the positive correlation of Moran’s I gradually increases with the continuous improvement of the coordinated development status. It indicates that the water, economy, and ecology of the CPUA are more coordinated, and there is a benign interaction and common development among the systems. The main reason for this is that technological progress and scientific and technological innovation in turn make the connection between these systems closer, enhance interdependence and synergy, and enhance regional ecological protection awareness, resulting in a more coordinated relationship between water utilization, economic and social development, and ecological environmental protection.

##### Local spatial autocorrelation

**Fig. 14:**
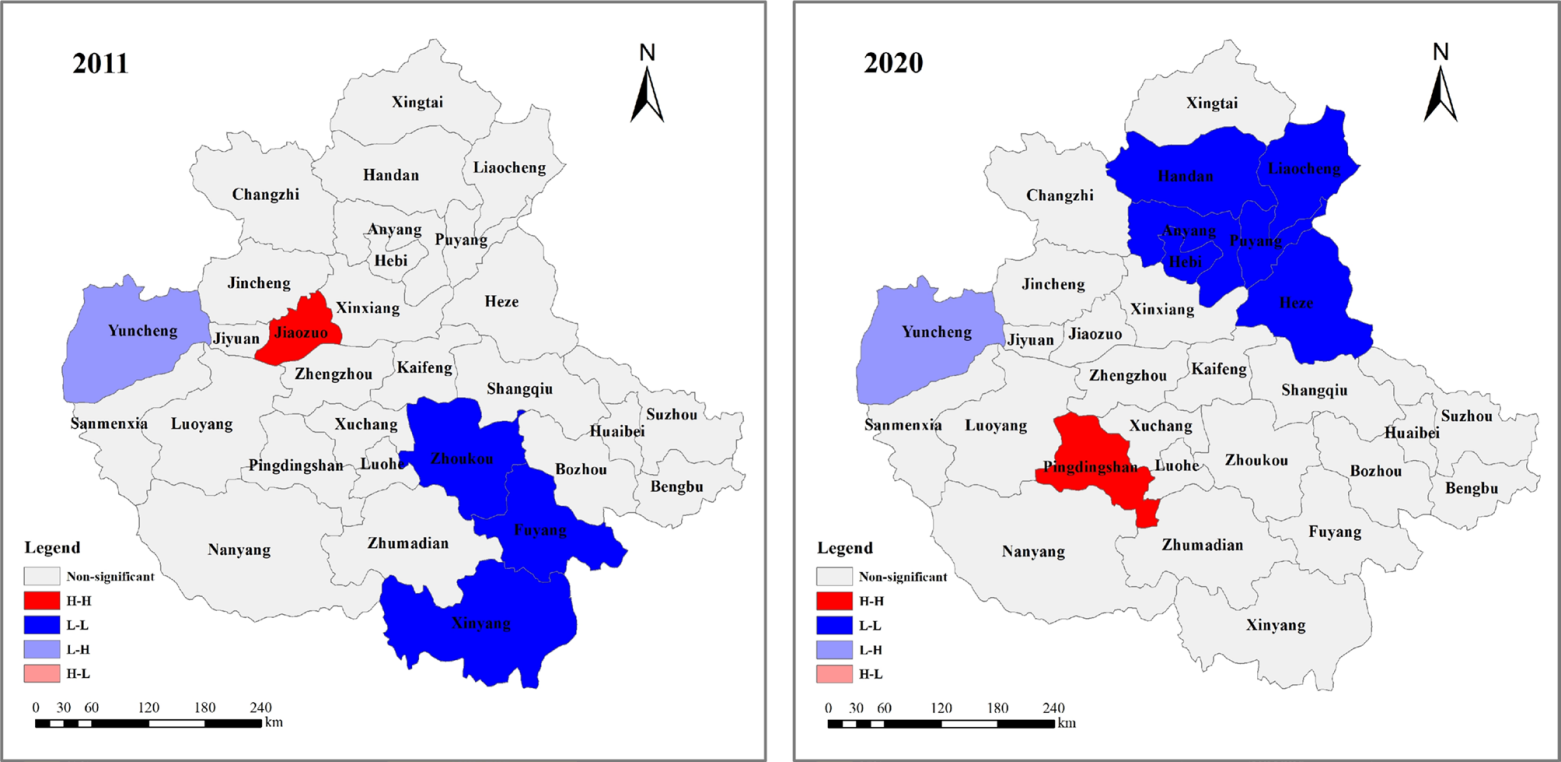
Local spatial autocorrelation clustering diagram of the CCD of WEE system in the CPUA (created using ArcGIS 10.8, https://www.esri.com, based on the standard map GS(2019)1822).

Since the value of Moran’s I reflects the estimation results of the whole area of the CPUA, it can only explain the overall spatial correlation of the WEE system’s CCD of the urban agglomeration, and cannot accurately reflect the local spatial characteristics. Therefore, in this section, Geoda software was used to test the local spatial autocorrelation. A K-nearest neighbors spatial weights matrix was constructed to ensure each county had an equal number of neighbors and to avoid the “island” problem caused by irregular boundaries, and the LISA cluster maps shown in Fig. 14 were drawn to carry out spatial visualization analysis.

As can be seen from Fig. 14, in 2011, the CCD of the CPUA shows L–H aggregation relationship for Yuncheng at the confidence level of p = 0.001; Jiaozuo shows H–H of p = 0.01; at the confidence level of p = 0.05, Xinyang, Zhoukou, and Fuyang show L-L aggregation relationship, and the rest of the 25 cities are insignificantly related to the relationship. In 2020, at the confidence level of p = 0.01, Handan is in L-L aggregation relationship; at the confidence level of p = 0.05, Pingdingshan is in H–H aggregation relationship, Anyang, Hebi, Puyang, Liaocheng, and Heze are in L-L aggregation, Yuncheng is in L–H aggregation relationship, and the remaining 22 cities are in non-significant correlation. In order to clarify the changes in the number of cities in each cluster relationship in the urban agglomeration during the period 2011–2020, Table 5 was organized based on the clustering results.

**Table 5:** Cluster aggregation relationship of CCD of WEE system in the CPUA.

Aggregation relationship	2011	2012	2013	2014	2015	2016	2017	2018	2019	2020
H–H	1	1	0	0	1	0	1	1	1	1
L-L	3	1	1	1	1	1	4	1	5	6
H–L	1	1	1	2	1	2	1	1	1	1
L–H	0	1	1	1	1	1	0	0	0	0

As can be seen from Table 5, the total number of L-L and H–L agglomeration cities dominates. The high number of L-L agglomeration cities implies that the urban agglomeration still has relatively large room for improvement, and that it needs to strengthen the coordination and integration of these aspects to enhance the sustainable development. The dominance of H–L agglomeration indicates that the development of the three subsystems is not balanced, and that the level of integrated management needs to be strengthened to realize the coordinated of the economy, environment and society. In addition, the number of cities with correlation shows an increasing trend, until 2020, there are already 8 cities with correlation, which means that the connection and influence of the cities in resources, environment, economy, and other aspects are gradually enhanced, and the urban agglomeration radically improves the closeness of the connection between the cities through the means of resource sharing, economic interaction, ecological and environmental prevention and joint management.

#### Study of CCD spatial linkages based on gravity model

**Fig. 15:**
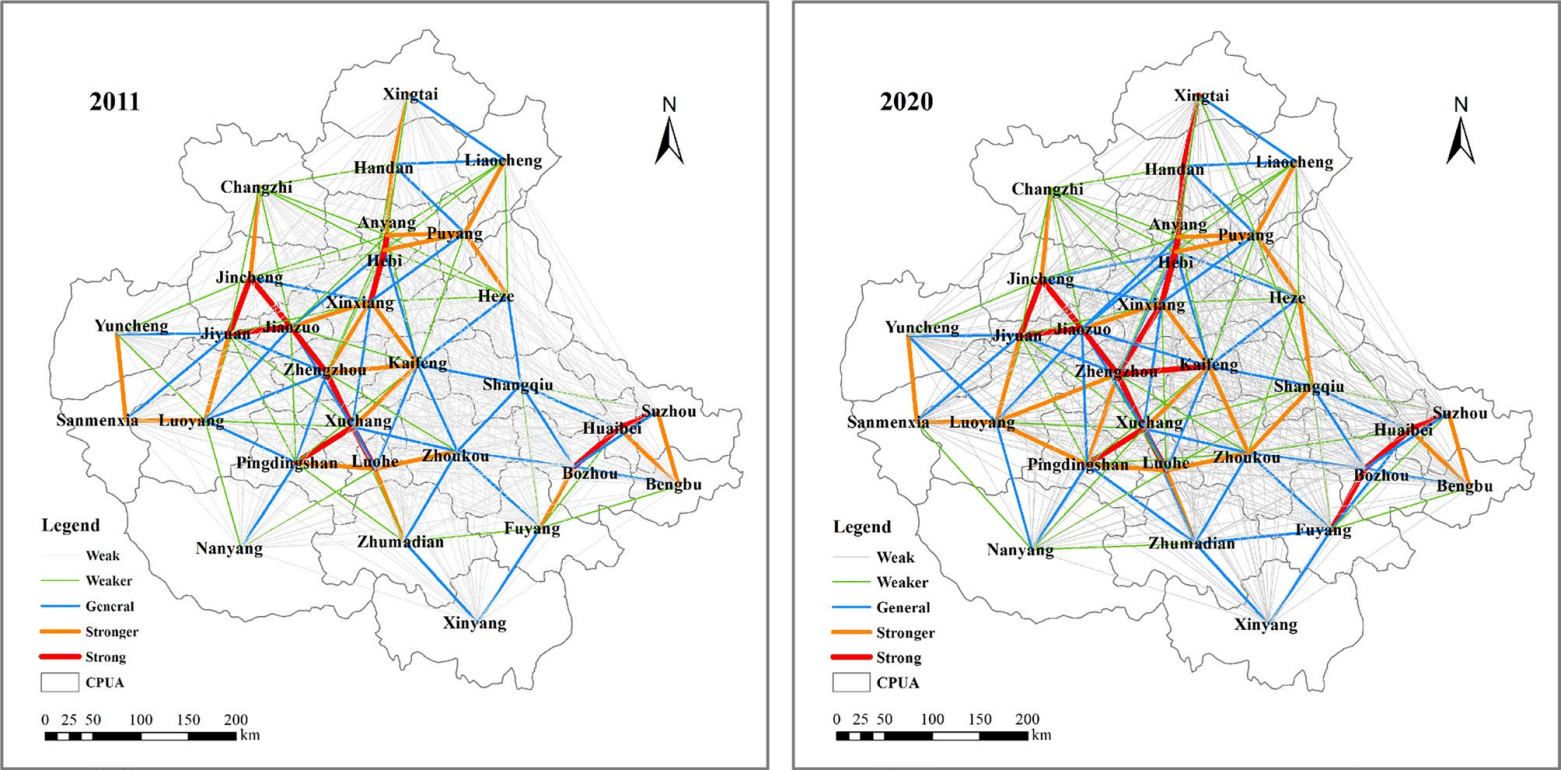
Strength of CCD spatial correlation of the WEE system in the CPUA in 2011 and 2020 (created using ArcGIS 10.8, https://www.esri.com, based on the standard map GS(2019)1822).

In this study, 2011 and 2020, the beginning and end of the study sequence, were selected as representative years, and the CCD gravitational force of the WEE system between 30 cities in the CPUA was calculated based on the gravity model, and then the spatial linkage strength between these cities was obtained by using the XY to line point-to-line function of ArcGIS, and Fig. 15 was plotted, so as to analyze the evolution of the spatial pattern of the CCD.

From Fig. 15, it can be seen that from 2011 to 2020, the spatial linkage strength of the CPUA has greatly improved. In terms of the value of city links, the total number of links in the CPUA in 2011 was 51.44, with an average link value of 0.12, and the total number of links in 2020 increases to 67.21, with an average link value of 0.15, which is a 30.7% increase in the value of links in the CPUA and an enhancement of the strength of spatial links. In 2011, the highest value of city linkage intensity was about 1412 times the lowest value, and in 2020, the multiple of the highest value and the lowest value of city linkage intensity decreased to 1319 times, and the polarization phenomenon was further reduced. In terms of the grading of city linkage axes, there are 435 linkage axes in the CPUA, with 11 strong linkage axes in 2011, accounting for 2.5%, 23 stronger linkage axes, accounting for 5.3%, 35 general linkage axes, accounting for 8.0%, 40 weaker linkage axes, accounting for 9.2%, and 326 weak linkage axes, accounting for 74.9%. Compared with 2011, in 2020, the number of strong linkage axes in the CPUA increases to 17, with a 1.4% increase, indicating that the CPUA is more closely spatially linked.

While the strength of the spatial linkage of the CCD of the CPUA has increased, the results of the calculation and the characteristics of the hierarchical distribution of city linkage axes still reflect some problems in the spatial linkage. For example, the spatial linkage is not balanced, weak linkage is still common, showing obvious “core–edge” structural characteristics, and the polarization phenomenon is more obvious. Zhengzhou has a prominent central position, but its radiation effect is limited. The average linkage strength between Zhengzhou and other cities in 2020 is 0.61, while the average linkage strength between cities in the entire CPUA is only 0.15, which indicates that Zhengzhou has a stronger radiation ability, but it mainly maintains a strong linkage with the nearby cities, and the linkage with other cities in the urban agglomeration is not strong.

#### COG migration of CCD

**Fig. 16:**
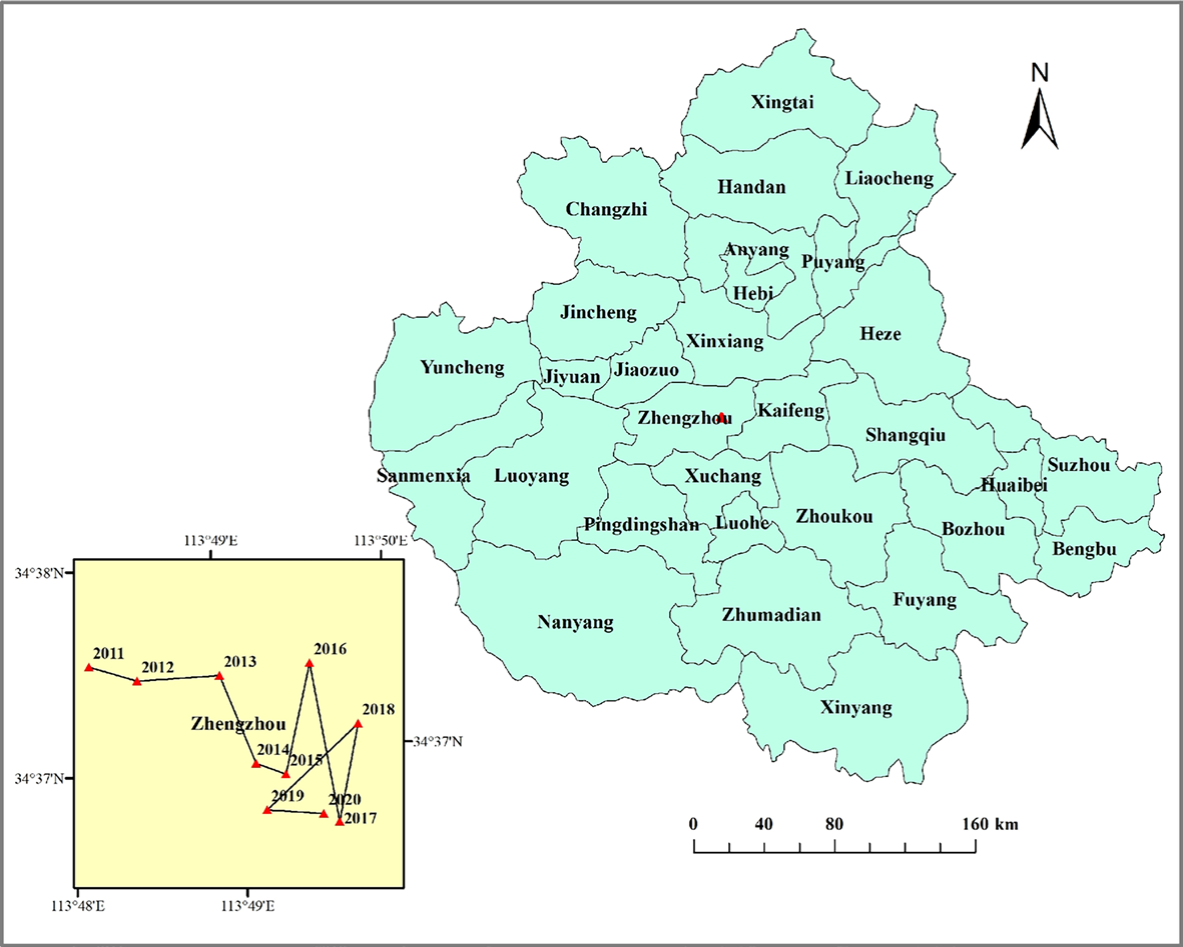
Trajectory of COG migration for CCD in the CPUA, 2010–2020 (created using ArcGIS 10.8, https://www.esri.com, based on the standard map GS(2019)1822).

In this study, the COG migration model was used to calculate the trajectory of the COG evolution of the CCD in the CPUA from 2011 to 2020, and Fig. 16 was plotted.

As can be seen from Fig. 16, the COG of the CCD in the CPUA during the study period is located in the Zhengzhou, with the geographic coordinates of the COG ranging from 113°48′E to 113°50′E and 34°36′N to 113°38′N. From the offset distance, the CPUA is offset by a total of 12.47 km from 2011 to 2020, with an average annual offset distance of 1.39 km, which makes the COG offset a smaller distance and the overall pattern more stable. The COG of the CCD has a larger offset distance in 2015 and 2016, respectively 1.90km and 2.74km.

The COG shift of the CCD can be divided into three stages: the first stage is 2011–2014, the COG of the CCD continued to shift to the southeast, during which a total of 1.59km was shifted, with an average annual shift distance of 1.09km. During the period of 2011–2013, the COG shifted at a relatively stable speed of 1.30 km/a, with a total shift of 3.90km, and in 2014, the shift tended to be slower, with a total shift of 0.45km, and the COG shifted at a rate characterized by the change of “stable-slow.” The second stage is 2015–2018, the COG shows a relatively large back and forth movement trend, during which it is shifted twice to the northeast, once to the southwest, and once to the southeast, and the southeast is shifted to the largest distance, with a movement speed of 1.99 km/a, and a total of 7.97km, and the COG offset speed shows a “rapid” change feature. In the third phase, from 2019 to 2020, the COG is shifted to the southeast again, with a total shift of 0.15 km during the period, and the COG is shifted at a rate characterized by a “slow” change.

Overall, the COG of the CCD of the CPUA moves towards the southeast as a whole from 2011–2020. The reason for this is that, in terms of the water system, the southeast has better water resource endowment, while the northwest is relatively poor, and climate change has made this major feature more pronounced, with the COG of water resources moving towards the southeast. From the perspective of economy system, with the development of integration in recent years, Kaifeng, as an emerging sub-center city, provides more space for Zhengzhou’s industrial development, and also brings a broad space for its own economic development, and Kaifeng’s backwardness causes the COG of the economy to migrate to the southeast. From the perspective of the ecology system, since “vigorously promoting the construction of ecological civilization” was put forward in 2012, Xuchang and other places have actively responded to the national policy, and achieved remarkable results in the field of ecological civilization, which led to the migration of ecological COG to the southeast.

## Discussion

### Research advantages and reference significance

In addition to being essential natural resources for human survival, water resources also constitute crucial economic resources that drive social and economic progress. As such, the coordinated development of the WEE system represents not only an ecological and environmental issue but also a matter of economic, social, and political significance that relates to national security. Within the field of water resources research, the CCD of the WEE system has become a prominent topic of study. The present research demonstrates the following advantages and reference value compared to previous studies.

#### Evaluation index system

Against the backdrop of the CPUA Development Plan and the overarching strategy for coordinated economic and environmental development, improving human living standards and the pursuit of better living conditions have drawn increasing attention to the coordinated development of the WEE system. However, there remains no unified standard for for defining the CDI of water, economy, and ecology, nor consensus on which indicators should be used for its measurement.

The establishment of an evaluation index system is essential for comprehensively understanding and assessing the development status of a given field. Given that the WEE system is a composite system involving a wide range of indicators, it is impractical to include all possible variables in the evaluation. Thus, the selection of the most relevant and targeted indicators becomes particularly important. In this study, strict adherence to established principles of index system construction was maintained, and scientific methods—such as frequency statistics^26^, gray correlation analysis^27^, and factor analysis^28^ were employed to screen a broad set of potential indicators. As a result, an evaluation index system encompassing three criterion layers (water, economy, and ecology) was constructed, comprising a total of 25 indicators.

Water serves not only as a driving force for economic development but also as a vital component for maintaining ecological balance, making it a critical element within the WEE system. Existing studies on WEE coupling often focus narrowly on attributes intrinsic to each subsystem, selecting indicators such as science and technology expenditure^29^, revenue from telecommunication services^30,31^, Number of inbound tourists^4^, and total retail sales of consumer goods^9–11^. However, many of these indicators exhibit limited or no inherent connection to water resources. This study not only applied rigorous methodological screening and considered the inherent characteristics of the three subsystems but also emphasized the interlinkages among them. Indicators were selected based on their relevance to water, thereby enabling a more accurate evaluation of the coupled development within the WEE system and facilitating targeted policy recommendations. The evaluation index system developed in this study may provide a valuable reference for research on the WEE system in other regions.

#### Evaluation methodology

Subsystem weights play a critical role in the calculation of the CCD, yet there remains no universally accepted method for weight allocation in composite systems. Weight determination methods are generally categorized into subjective and objective approaches. Among subjective methods, the analytic hierarchy process (AHP) is widely adopted and has been extensively applied and validated in numerous studies for its feasibility^23,24,32,33^. Objective weighting methods commonly include entropy weighting^4,34^, CRITIC^9–11,35^, coefficient of variation^23,24,36^, and so on. With advancing research, an increasing number of studies have begun integrating both subjective and objective weighting strategies to enhance accuracy^37,38^.

In this study, a hybrid weighting approach combining subjective and objective methods was employed, utilizing both AHP and EFAST. This combination helps ensure that the resulting CCD values closely reflect real-world conditions. Compared to other objective weighting techniques, the EFAST method offers distinct advantages: it quantifies both the sensitivity of individual indicators to the results and the uncertainty inherent in their interactions. Luan et al.^39^ applied EFAST in a sustainable development evaluation and demonstrated that it provides richer information and greater specificity in quantitative analysis and comprehensive assessment compared to traditional methods.

The CCD of the WEE system is an important factor in maintaining the sustainable development of human beings. Evaluating the condition of the CCD of the WEE system objectively helps in developing precise water resource management strategies. Because the state of CCD is influenced by several circumstances, research on the CCD evaluation of the WEE system is often hindered by uncertainty and fuzziness. It is important to pay special attention to the sensitivity of evaluation indices and the fuzziness of threshold values.

To address these challenges, this study introduced a cloud model to evaluate the development level of each subsystem, and the result was used to derive CDI, thereby refining the traditional coupled coordination evaluation framework. The cloud model serves as a versatile tool for representing both qualitative and quantitative changes, capturing the uncertainty and imprecision of language notions while facilitating the conversion between numerical data and abstract ideas. Therefore, the uncertain problem of system development level evaluation can be properly solved, and it has a unique advantage in dealing with fuzziness and randomness. The approach provides an important reference and theoretical basis for weight calculation, comprehensive development evaluation, and multi-system coupling coordination measurement.

#### Evaluation path

Numerous studies on the CCD system have concluded that such systems consistently evolve toward coordination across various regions^30,31,40,41^, and this study aligns with that finding. However, due to variations in research focus, there are disparities in the elements impacting the coordinated growth of the system as well as in the development and changes of subsystems. Many previous studies have overlooked the analysis of the CDI of subsystems, which is crucial for promoting regional coordinated development. This study addresses this gap by evaluating and analyzing the development levels of the three subsystems and the overall WEE system before assessing the CCD, thereby establishing a solid foundation for understanding regional comprehensive growth and conducting CCD evaluation.

Using the ECM and ICCDM, this study examined the coupling relationships among the water system, economy system, and ecology system in the CPUA, as well as their temporal and spatial heterogeneity. To further clarify the level of coupling coordination between these systems, the CCD of dual systems was measured and analyzed from both the perspective of the urban agglomeration as a whole and the multi-year average for each city. Subsequently, the CCD of the WEE system was evaluated and its spatiotemporal evolution was analyzed at three scales: the urban agglomeration, urban partitions, and individual municipalities. This multi-scale and multi-perspective approach offers a clearer understanding of the coupled and coordinated development of regional composite systems and provides important theoretical support for future development strategies.

Urban agglomerations are characterized by three key features: a certain number, high density, and diversity of city types; the presence of one or more megacities as cores; and strong intrinsic interconnectedness among cities. Therefore, analyzing the spatial relationships of CCD within urban agglomerations is particularly important. In this study, spatial autocorrelation of CCD in the CPUA was analyzed using Moran’s I and LISA cluster maps. Furthermore, based on the gravity model,the gravitational effect of CCD between each city in the urban agglomeration of 2011 and 2020 and its evolution were calculated. Finally, the COG model was applied to identify the annual location and migration trajectory of the CCD centroid during the study period. These three steps together provide a systematic analysis of the spatial relationships underlying CCD within the urban agglomeration.

This study adopted an evaluation pathway encompassing comprehensive system evaluation, coupling coordination degree assessment, and spatial relationship analysis. By employing diverse methods and multi-scale perspectives, it offers a structured approach that can serve as a reference for CCD evaluations in other urban agglomerations and multi-system coupling coordination studies.

### Future prospects

The data used in this study cover the period from 2011 to 2020. Although the COVID-19 pandemic significantly influenced China’s economic development between 2020 and 2023, the lack of comprehensive statistical data during this interval prevented an in-depth analysis. As we have now entered the post-pandemic era, characterized by substantial changes in economic conditions and public awareness, future work will aim to extend the research timeline to include pre- and post-pandemic comparisons. In future studies, it would be worthwhile to incorporate indicators such as drought index and effluent ion concentration, as well as to account for the impact of COVID-19 on the coordinated development of the WEE system. This will facilitate a clearer assessment of the pandemic’s impact on the CCD of the WEE system at the regional level.

This study focuses on evaluating the current state of the CCD within the WEE system in the CPUA and does not include projections of future trends. Subsequent research will incorporate theoretical approaches such as SD to simulate and predict the coordination development under various scenarios. These efforts are expected to offer theoretical guidance and support for sustainable urban planning and policy-making.

## Conclusions

Water, economy, and ecology are closely interconnected, collectively forming a WEE composite system. Investigating the coupling status of this composite system, analyzing its evolutionary patterns and spatial distribution, is of great significance for promoting its sustainable and coordinated development in the future. Taking the CPUA as the research area, this study scientifically constructed an evaluation index system by identifying key influencing factors of each subsystem and employing multiple methods for screening indicators. The CCD of the WEE system was measured and analyzed in terms of spatial and temporal variations. Furthermore, spatial autocorrelation, gravitational effects, and center of gravity migration were calculated to elucidate the spatial interrelationships among cities. The main conclusions are as follows.

1. Among the three subsystems of water, economy, and ecology, *A*_*1*_ (Per-capita water resource), *B*_*1*_ (Per-capita GDP), and *C*_*1*_ (Greening coverage in built-up areas) are the most heavily weighted indicators, with weights of 25.66, 22.34, and 19.75%, respectively.
2. From 2011 to 2020, the CDIs of the three subsystems and the WEE composite system exhibited a fluctuating upward trend. The ecological subsystem showed the most significant improvement, followed by the economic subsystem. The CDI of the WEE system was higher in the CDA than in the NCDA, and higher in the southern region compared to the northern region.
3. Although the CD and CCD of the dual system experienced some fluctuations, an overall upward trend was observed. By 2020, the CPUA was predominantly characterized by advanced Water-Ecology dual system coordination, while cities with advanced Water-Economy dual system coordination had largely disappeared, reflecting achievements in ecological civilization construction. The CD of the WEE composite system remained relatively stable over the years, and the CCD showed steady growth except for a decline in 2019 due to severe drought. Within the NCDA-E, the WEE system exhibited the highest level of coordination. Among individual cities, Jiyuan, Huaibei, and Zhengzhou had the highest average CCD values, reaching 0.8348, 0.8299, and 0.8127, respectively.
4. The global autocorrelation of CCD in the WEE system varied considerably but generally trended toward positive correlation. The local spatial autocorrelation was relatively weak. By 2020, only eight cities exhibited localized correlation, which is mainly dominated by “L-L” aggregation. From 2011 to 2020, the spatial gravitational strength of the CCD among the 30 cities in the CPUA strengthened noticeably, with the average linkage value increasing by 25%. The COG of CCD consistently remained within Zhengzhou, with an average annual displacement of less than 2 km, indicating overall regional stability.

## Data Availability

All data supporting the findings of this study are available within the paper.

## Code availability

This study did not use any custom code. All analyses were performed using available software.
